# The structure of caseinolytic protease subunit ClpP2 reveals a functional model of the caseinolytic protease system from *Chlamydia trachomatis*

**DOI:** 10.1016/j.jbc.2022.102762

**Published:** 2022-12-01

**Authors:** Jahaun Azadmanesh, Mohamed A. Seleem, Lucas Struble, Nicholas A. Wood, Derek J. Fisher, Jeffrey J. Lovelace, Antonio Artigues, Aron W. Fenton, Gloria E.O. Borgstahl, Scot P. Ouellette, Martin Conda-Sheridan

**Affiliations:** 1 The Eppley Institute for Research in Cancer and Allied Diseases, Fred & Pamela Buffett Cancer Center, University of Nebraska Medical Center, Omaha, Nebraska, USA; 2 Department of Pharmaceutical Sciences, University of Nebraska Medical Center, 986125 Nebraska Medical Center, Omaha, Nebraska, USA; 3 Department of Pathology and Microbiology, University of Nebraska Medical Center, 985900 Nebraska Medical Center, Omaha, Nebraska, USA; 4 School of Biological Sciences, Southern Illinois University Carbondale, Carbondale, Illinois, USA; 5 Department of Biochemistry and Molecular Biology, The University of Kansas Medical Center, Kansas City, Kansas, USA

**Keywords:** *Chlamydia*, ClpP, caseinolytic protease, crystal structure, HDX-MS, hydrogen-deuterium exchange mass spectrometry, PDB, Protein Data Bank

## Abstract

*Chlamydia trachomatis* (ct) is the most reported bacterial sexually transmitted infection worldwide and the leading cause of preventable blindness. Caseinolytic proteases (ClpP) from pathogenic bacteria are attractive antibiotic targets, particularly for bacterial species that form persister colonies with phenotypic resistance against common antibiotics. ClpP functions as a multisubunit proteolytic complex, and bacteria are eradicated when ClpP is disrupted. Although crucial for chlamydial development and the design of agents to treat chlamydia, the structures of ctClpP1 and ctClpP2 have yet to be solved. Here, we report the first crystal structure of full-length ClpP2 as an inactive homotetradecamer in a complex with a candidate antibiotic at 2.66 Å resolution. The structure details the functional domains of the ClpP2 protein subunit and includes the handle domain, which is integral to proteolytic activation. In addition, hydrogen-deuterium exchange mass spectroscopy probed the dynamics of ClpP2, and molecular modeling of ClpP1 predicted an assembly with ClpP2. By leveraging previous enzymatic experiments, we constructed a model of ClpP2 activation and its interaction with the protease subunits ClpP1 and ClpX. The structural information presented will be relevant for future rational drug design against these targets and will lead to a better understanding of ClpP complex formation and activation within this important human pathogen.

*Chlamydia trachomatis* (ct), an obligate intracellular Gram-negative bacterium, is the primary bacterial sexually transmitted disease and cause of preventable infectious blindness (trachoma) worldwide (1, 2). Great efforts have tried to develop antimicrobials that can control and halt the rate of *C. trachomatis* infections (3, 4, 5, 6). *Chlamydia* undergoes a developmental cycle transitioning between an infectious elementary body and a replicative reticulate body (7). The elementary body and reticulate body forms have distinct proteomes, suggesting that protein turnover is critical to the development of the pathogen (8, 9). Caseinolytic proteases (Clp) are a family of conserved multiple-subunit enzymes found in bacteria and higher organisms that have become an attractive and novel therapeutic target to combat infection (10, 11). Either activation (11, 12) or inhibition (13, 14) of the ClpP protease component can disrupt the proteolytic process and eradicate the pathogen, including biofilms and persister cells (15). Targeting the chlamydial Clp components has reduced the chlamydial burden in cell culture and animal models of infection (16, 17, 18).

ClpP assembles into tetradecameric barrels consisting of two heptameric rings. These barrels interact with hexameric AAA+ unfoldases, such as ClpX, ClpA, or ClpC, to unfold the targeted protein and facilitate its entrance into the proteolytic barrel. The first identified and fully characterized ClpP was from *Escherichia coli* (ec), a known critical factor for growth and development (19, 20, 21). While most bacterial genomes possess a single copy of *clpP*, some pathogenic bacteria, including *Mycobacterium tuberculosis*, *Listeria monocytogenes*, and *Pseudomonas aeruginosa*, have two copies of *clpP* genes (18, 22, 23, 24, 25). These ClpP1/P2 complexes’ functional arrangement depends on the species, ranging from homotypic heptamers, inactive homotypic tetradecamers, active homotypic tetradecamers, or active heterotypic tetradecamers (26, 27, 28, 29). Intriguingly, *Chlamydia*, an organism with a highly reduced genome, encodes two ClpP orthologs (*clpP1* and *clpP2*) (18, 30) and possesses two unfoldases: *clpX* and *clpC*. Interestingly, *clpP2* is present in an operon with the unfoldase *clpX*, suggesting ClpP2 may form an active complex with ClpX, whereas *clpP1* is present at a discrete locus on the chromosome and independently of *clpC*.

The nature of oligomerization, the 3D arrangement of the protein, and the dynamics of the functional domains of the *Chlamydia* ClpP orthologs remain to be clarified. Our previous work suggested the *Chlamydia* ClpP orthologs can function independently of one another based on differential effects when expressed *in vivo* (17, 18). We also observed *in vitro* proteolytic activity of recombinant WT ClpP2 but no activity of its inactive isoform (S98A) or the ClpP1 protein (18). However, a subsequent study using protein preps prepared from an *E. coli* strain lacking its Clp proteases (*i*.*e*., Δ*clpPAX*) observed *in vitro* activity of heterotypic ClpP1P2 protease complexes but none for either ortholog alone (30). These data would suggest the activity detected only in our WT ClpP2 preps may have been caused either by small amounts of contaminating, co-purified *E. coli* ClpP or by *E. coli* ClpP processing the chlamydial isoform or otherwise providing some signal, to render it active. Regardless, it remains unclear the specific composition of active Clp complexes *in vivo*. As part of the present study, we initiated a structural assessment of the *C. trachomatis* ClpP2 protein (ctClpP2) to discern the structural mode of oligomerization, providing a foundation for rational drug design and clues to the potential *in vivo* function of this protein.

Like other ClpPs, ctClpP2 is presumably part of the proteolytic tetradecamer where a ctClpP2 heptamer may oligomerize with another ctClpP2 heptamer to form a homotetradecamer or with a ctClpP1 heptamer to form a heterotetradecamer (31, 32, 33, 34, 35). The tetradecameric arrangement sequesters the catalytic triad of each monomer within its barrel. Proteolytic activity requires alignment of the catalytic triad through conformational changes of the handle domain that composes the interface between heptamer rings. The inactive tetradecamer is represented by triad residues that are not aligned and thus incapable of proteolysis. Alignment requires association with an AAA+ unfoldase like ClpX (36), at the hydrophobic groove of ClpPs formed by intercalation between two adjacent monomers. The unfoldase-binding site is an attractive target for developing antibacterial drugs. For example, small molecules like the acyldepsipeptides (ADEPs) and ACPs (activators of cylindrical proteases) are typically ClpP activators that compete with the AAA+ unfoldase to bind ClpP, typically leading to alignment of the catalytic triad (37). In some species, such as *M. tuberculosis*, these antibacterial drugs are incapable of activating ClpP, and a second allosteric stimulus like dipeptide-binding adjacent to the catalytic triad is required (38). Therefore, we pursued a detailed understanding of the ctClpP2 structure and activation to facilitate the design of specific molecules for this protein and, presumably, *Chlamydia*.

Herein, we present a model of ctClpP2 activation and interaction with ctClpP1 and ctClpX. First, we verify the homotetradecameric state through atomic force microscopy, transmission electron microscopy, and crystallography. Second, we investigated the binding of an ACP-like ligand, MAS1-12, to ctClpP2 by solving the crystal structure at 2.66 Å (16). The crystal structure is noteworthy in that the complete functional domains key for activity and the candidate antibiotic that is bound were only visualized in the electron density maps when the crystallographic data were carefully analyzed and then treated for pseudomerohedral twinning in the lowest symmetry space group, P1. Several ClpP structures in the Protein Data Bank (PDB) lack density in these regions and are not in the atomic model. We suspect that previous homologous ClpP structures lacking structure for the functional domains could have been pseudomerohedrally twinned and possibly solved in an inappropriately high symmetry space group. The complete full-length ctClpP2 structure solved had notable differences in the arrangement of its functional domains compared to ClpPs from other bacterial species. Third, we investigate the conformational dynamics of the homotetradecamer with hydrogen-deuterium exchange mass spectrometry (HDX-MS). Finally, we interweave our crystal structure with *a priori* information from ClpPs of other species to construct a model of activated ctClpP2 and ctClpP1. We expect this investigation will provide valuable information for the design of compounds that selectively target either of the proteins, to understand the mode of catalytic activation and complex composition, to study putative interacting partners, and to investigate proteolytic activity during the developmental cycle of *Chlamydia*.

## Results

### Crystal structure of inactive ctClpP2

**Figure 1:**
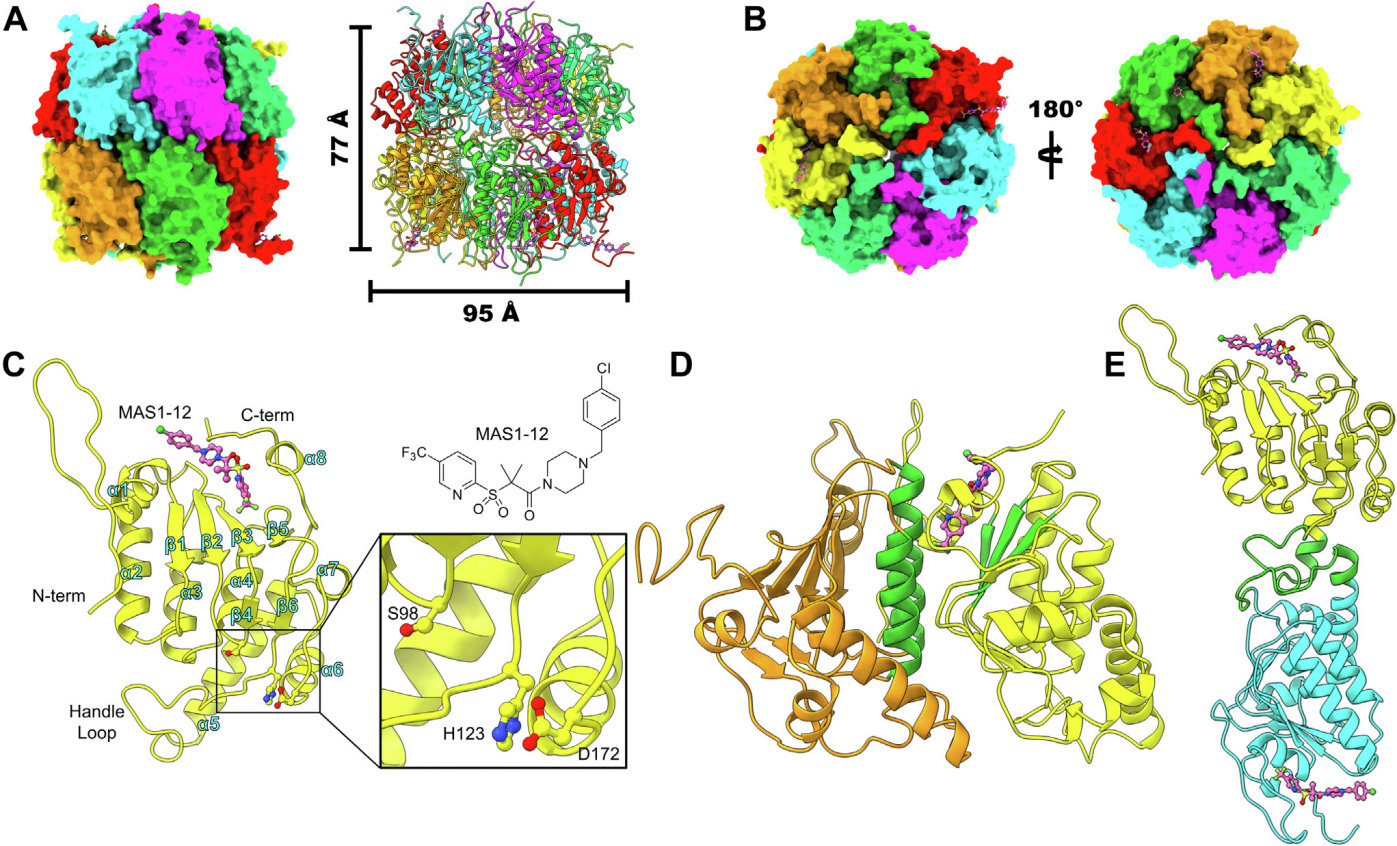
**Crystal structure of ctClpP2.***A*, structure of the tetradecameric ctClpP2 asymmetric unit illustrated as surfaces and ribbon drawing. *B*, the axial surfaces of the ctClpP2 tetradecamer. *C*, monomeric structure of MAS1-12–bound ctClpP2. The catalytic triad is not aligned and therefore inactive. Secondary structure motifs are labeled in *cyan* lettering and numbered independently by type starting from the N terminus. *D*, the ctClpP2 heptamerization interface. Oligomerization into heptamers entails aligned α-helices of one subunit interfacing with the aligned β-sheets of another shown in *green*. *E*, the ctClpP2 tetradecamerization interface. Oligomerization of heptamers into tetradecamers involves the handle domains of monomers from opposing heptameric rings interdigitating as shown in *green*.

The asymmetric unit of ctClpP2 includes two opposing heptameric rings that form a tetradecamer with a height of 77 Å and a diameter of 95 Å (Fig. 1, *A* and *B*). Transmission electron microscopy images show a 7-sided ring with a central pore, while atomic force microscopy shows a height that is consistent with two rings forming a barrel (one on top of the other) or a homotetradecamer (Fig. S1). The monomers in the crystal structure follow the canonical ClpP fold with the secondary structure consisting of a set of aligned α-helices (α2- α5, Fig. 1*C*) behind four β-sheet motifs aligned in the direction of the α-helices (β1-β3 and β5, Fig. 1*C*) (38, 39, 40, 41). The heptameric interfaces consist of aligned α-helices of one subunit interfacing with the aligned β-sheets of another in a front-to-back interaction (Fig. 1*D*). The tetradecameric interface is composed of handle domains from opposing heptamers interlocking (Fig. 1*E*).

The handle domain of ctClpP2 (and other ClpPs) is responsible for aligning the catalytic triad through conformational changes involving the N-terminal and C-terminal domains. Conformational changes are propagated by binding the ClpX unfoldase, small molecule inhibitors/activators, or dipeptide (36). These domains have been historically difficult to resolve for the inactive form of ClpPs due to their disorder (42). However, despite the modest 2.66 Å resolution, residues of the handle and terminal domains were distinguished by electron density for a majority of the subunits (Fig. S2). Surprisingly, we observed residues Ser98, His12, and Asp172, which compose the catalytic triad, unaligned while ctClpP2 is bound by MAS1-12 (Fig. 1*C*). Lack of hydrogen bonding among the triad indicates that the ctClpP2 residues are in an inactive form and that a conformational change is required for the catalytic residues to interact (38, 41). Indeed, we previously demonstrated that MAS1-12 is capable of activating ecClpP on its own but not ctClpP2 or ctClpP1 (16). This observation is consistent with the activation mode of the ClpP from *M. tuberculosis*, where small molecule binding alone is not sufficient for triad alignment and proteolytic activity; dipeptide binding at the handle domain is required (38).

### Distinguishing features of *C*. *trachomatis* ClpP2

**Figure 2:**
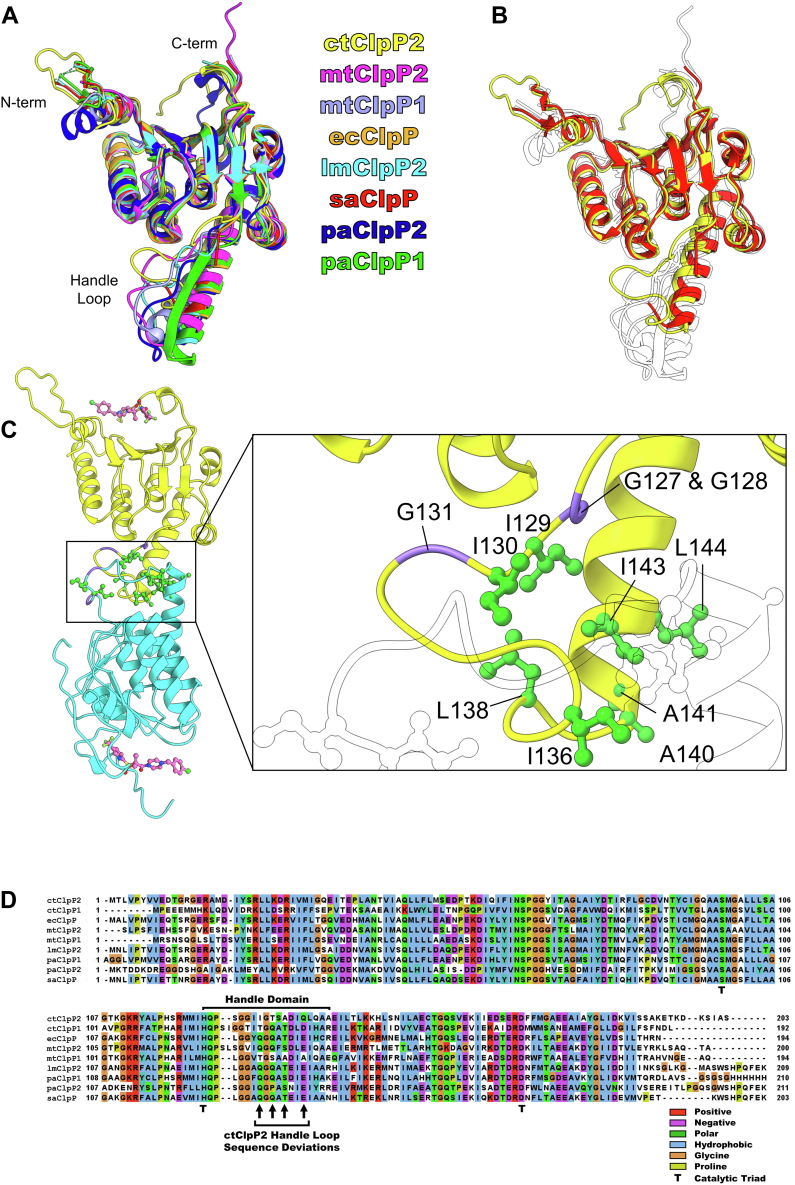
**Contrasting structural features of ctClpP2 compared to homologs.***A*, structural comparison of the ctClpP2 inactive monomer to other inactive homologs. *B*, same as (*A*) homologs ctClpP2 and saClpP that have a shorter, compact handle domain are highlighted in *yellow* and *red*. *C*, the hydrophobic packing at the handle domain with hydrophobic residues highlighted in *green* and the protein backbone of glycine residues colored *purple*. *D*, multiple sequence alignment and analysis of ClpP homologs using *CLUSTAL OMEGA* for the alignment process and *JALVIEW2* for visualization (67, 68). Color coding indicates consensus residue type. Notable ctClpP2 deviations of consensus type within the handle loop are denoted by arrows.

Comparison of the ctClpP2 crystal structure with inactive ClpPs from other species reveals key defining features. We utilized the following ClpP structures for analysis: ClpP2 from *L. monocytogenes* (lmClpP2, PDB ID 4JCT) (39), ClpP from *Staphylococcus aureus* (saClpP, PDB ID 4EMM) (40), ClpP from *E. coli* (ecClpP, PDB ID 3HLN) (41)*,* heteromeric ClpP1/P2 *M. tuberculosis* (mtClpP1/P2, PDB ID 6VGK) (38), ClpP1 from *P. aeruginosa* (paClpP1, PDB ID 7M1M) (43), and ClpP2 from *P. aeruginosa* (paClpP2, PDB ID 7M1L) (43). First, ctClpP2 is the only ClpP among those compared that has both a flexible N terminus and a mini-helix, also called α8, at the C terminus (Figs. 1*C* and 2*A*). The N terminus determines the size of the axial pore and makes contact with the pore-2 loops of the ClpX/C unfoldase, whereas the C terminus mediates access to the hydrophobic pockets that the IGF/IGL loops of ClpX/C binds (33, 44). In particular, an N terminus with a flexible axial loop is a noted determinant of recognition and activation by ClpX for lmClpP2 and paClpP1 (43). Second, the handle loop is more compact and the α5 helix that follows is 2∼3 helical turns shorter in ctClpP2 (Fig. 2*B*). This means oligomerization leads to a ctClpP2 tetradecamer that is shorter in height, 77 Å, compared to counterparts from other species, > 85 Å, except for saClpP that also has a compact handle domain. Of note is that saClpP has been shown to undergo a large conformational change for activation, where the α5 helix gains 2∼3 helical turns (45). Inspection of the handle domain interactions between ctClpP2 subunits reveals hydrophobic clustering of isoleucine, leucine, and alanine residues where one subunit loop is more coiled and compact than the other across the interface (Fig. 2*C*). The shorter handle domain of ctClpP2 may be explained by differences in residue composition that increase hydrophobicity. For example, ctClpP2 has fewer polar and ionic residues than the other ClpPs listed in Figure 2 and is the only ClpP that contains three isoleucine residues (Fig. 2*D*). These distinguishing features of ctClpP2 at the terminal and handle domains are presumed to affect catalytic activation and the interaction with the ClpX/C unfoldase.

### HDX-MS

The shorter and more hydrophobic handle domain of ctClpP2 compared to other homologs led us to investigate its flexibility. ClpP flexibility is integral to its function because it reflects the amenability of conformational change upon unfoldase (or small molecule) binding. To probe the dynamics of ctClpP2, we performed HDX-MS. HDX-MS measures the amount of deuterium exchange at the amide backbone for a hydrogenated protein in D_2_O and reflects solvent accessibility and hydrogen bonding participation. For highly flexible portions of ctClpP2, higher rate of exchange is expected.

**Figure 3:**
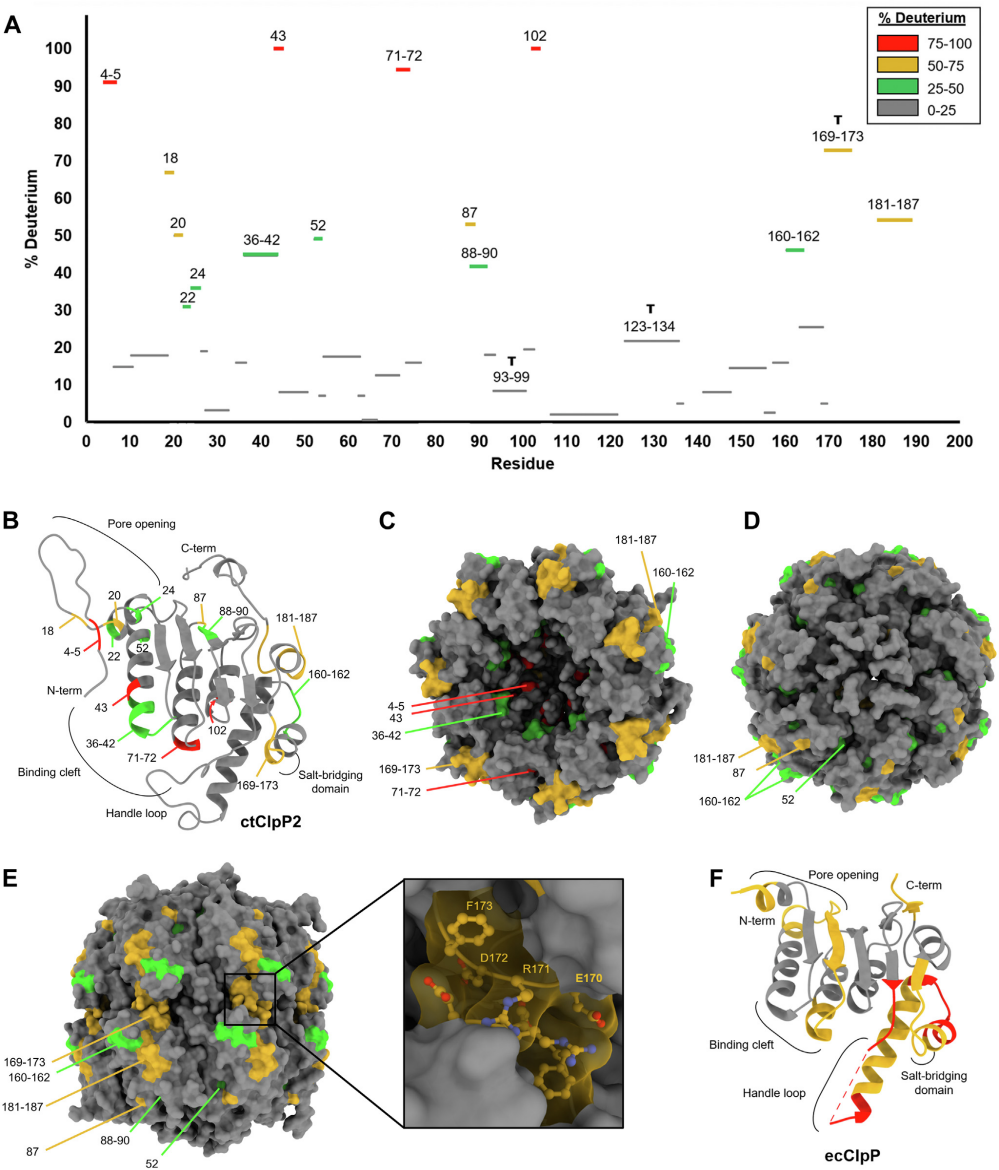
**Hydrogen-deuterium exchange mass spectroscopy (HDX-MS) of ctClpP2.***A*, percent deuterium of amide backbone protein fragments after 8 h of incubation with D_2_O. Fragments with catalytic triad residues Ser98, His123, or Asp172 are marked with a T. Fragments are colored by quartiles of percent deuterium designated in the graph. *B*, mapping of the hydrogen-deuterium exchange data onto the ctClpP2 monomer, (*C*) the inner pocket surface of the heptamer, (*D*) the apical surface of the heptamer, and (*E*) the surfaces of the tetradecamer with a zoom of region 169 to 173. Color coding reflects the percent deuterium quartiles seen in (*A*). *F*, HDX-MS mapping onto a monomer of ecClpP using PDB ID 3HLN (41) and data from Sowole *et al*. (31). The overall sequence coverage from HDX-MS measurements of ctClpP2 and ecClpP were 85% and 95%, respectively. PDB, Protein Ddata Bank.

After 8 h of D_2_O incubation, the peptide fragments of ctClpP2 measured by mass spectrometry indicate three regions with deuterium exchange above 90% (Fig. 3*A*). Earlier time points were not more informative in regard to protein flexibility (Fig. S3).The first includes residues Val4 and Pro5 that are part of the N-terminal loop and expected to be involved in axial pore opening during interaction with ClpX (Fig. 3*B*) (32, 46). Residues with moderate exchange rates near this region, Met18, Ile20, Ser22, Leu24, and Met52, may act as a flexible hinge to mediate pore opening. The second region consists of residues Thr43, Ile71, and Thr72 that are bridged by residues 36 to 42 with moderate exchange rates. These residues make the cleft within the inner pocket of the tetradecamer (Fig. 3*C*). Comparison with the ecClpPX and lmClpXP1/2 cryo-EM structures suggests this region is the putative ClpX IGF-loop binding site that coincides with pore opening (32, 33). Similarly, structures of drug-bound ClpP in the active conformation from other species indicate the region is the site of binding for ADEPs and ACPs (46). While these putative sites are not found on the apical surface (Fig. 3, *C* and *D*), it is expected that the opening of the pore reveals them. It should be noted that the present ctClpP2 crystal structure is drug bound but in an inactive form. The third region consists of residue Leu102 that is mostly buried in the crystal structure, but flexibility at this location may reflect the need for Ser98 to align with other members of the triad for catalytic activity (Fig. 3*B*). Taken together, the locations of higher rates of deuterium exchange correspond with prospective sites of conformational change or external binding.

Levels of intermediate deuterium exchange are observed for residues 169 to 173, including catalytic triad residue Asp172 (Fig. 3*A*). These residues are known to undergo a conformational change during proteolytic activation (38). For the inactive form observed in the crystal structure, the backbone loop on which amino acids 169 to 173 reside is compacted against the same residues from opposing monomers. This allows residues Glu170 and Arg171 to be solvent exposed (Fig. 3*E*). In the activated form, the backbone loops between monomers extend away from each other and coincide with the formation of electrostatic bridges between Glu170 of one monomer and Arg171 of the other (38). This movement is expected to contribute to aligning Asp172 with other members of the triad.

To compare the flexibility of ctClpP2 with another homolog, we obtained published HDX-MS data for ecClpP without activators (Fig. 3*F*) (31). A comparison of the HDX-MS data between ctClpP2 and ecClpP indicates drastic differences in flexibility. For ecClpP, the entirety of the handle loop, including the His of the triad, has deuterium exchange above 80% while ctClpP2 has exchange below 25% (Fig. 3, *B* and *F*). This may be reflected in the crystal structures, where the ecClpP handle domain was unable to be resolved due to disorder while the rigidity of the ctClpP2 handle loop contributed to a well-ordered structure that could be resolved by electron density. Similarly, a large portion of the ecClpP protein following the handle loop has higher exchange rates, including the areas flanking the region expected to undergo salt bridge extension for activation. Notably, there is flexibility linking the pore opening and binding cleft regions for ecClpP that is not seen in ctClpP2, which may have implications for activation. In general, the HDX-MS data suggest that ctClpP2 is much more rigid than ecClpP and provide a potential explanation as to why MAS1-12 activates ecClpP and not ctClpP2 (16).

### Binding mode of MAS1-12 to ctClpP2

**Figure 4:**
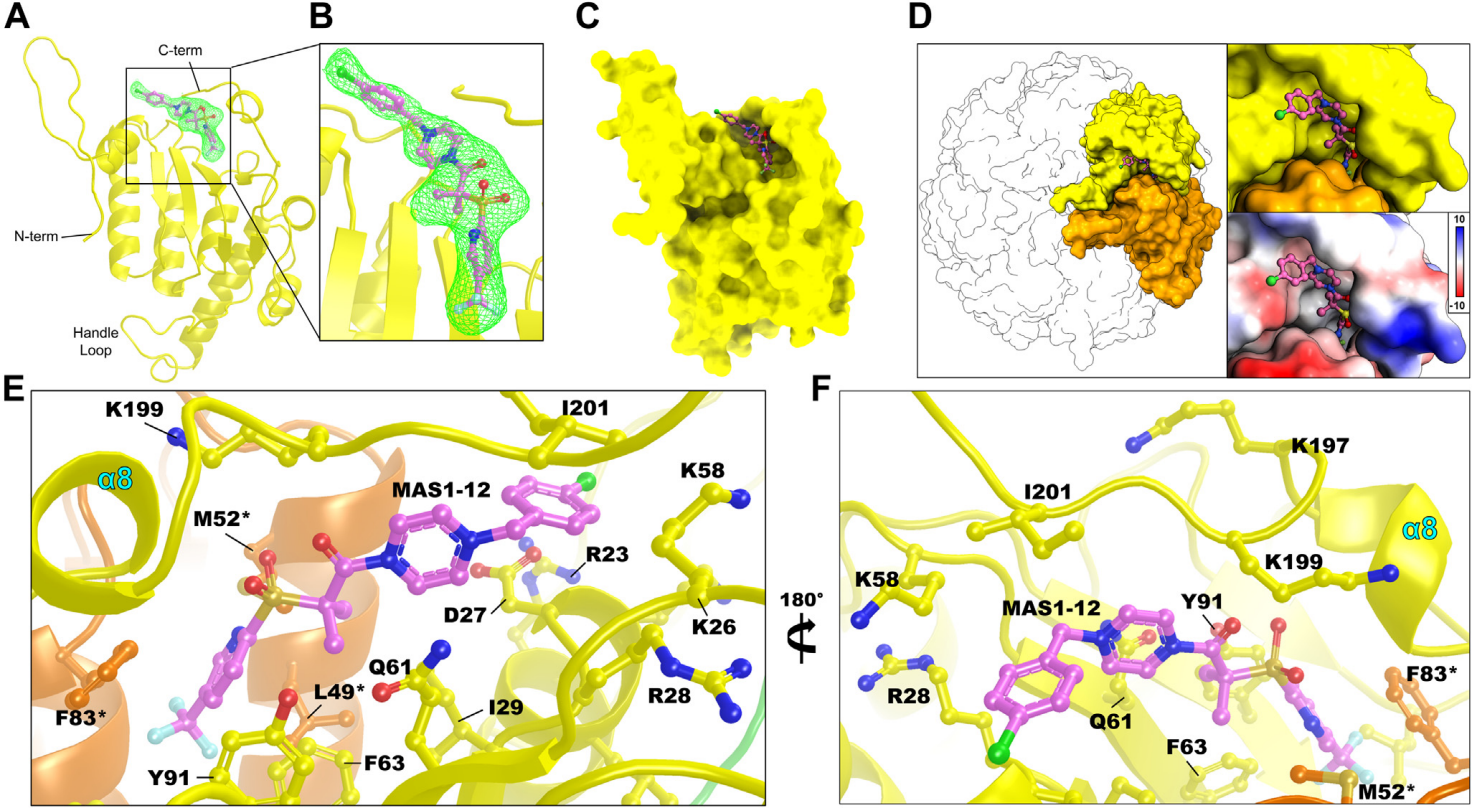
**The binding mode of the ACP-like ligand MAS1-12 to ctClpP2.***A* and *B*, omit |*F*_o_| - |*F*_c_| difference density contoured at 3.0 σ indicating binding of MAS1-12. *C*, depiction of the hydrophobic binding pocket that MAS1-12 binds in the context of the ctClpP2 monomer. *D*, depiction of the hydrophobic binding pocket in the context of two adjacent monomers with the inset noting electrostatic surface potentials in units of kcal/(mole·*e*). The *yellow* monomer in (*D*) is rotated 90° about the horizontal axis relative to (*C*). *E* and *F*, detailed zoom-in of the MAS1-12–binding pocket. Labels with asterisks indicate residues of the *orange* monomer. The Cl atom is colored *green* and F atoms are colored *light blue*.

To understand how a small molecule may bind to the ctClpP2, we crystallized the protein with an ACP ligand. MAS1-12 shares structural similarities with ACP compounds known to bind ecClpP and has been shown to activate that protease but not ctClpP2 or ctClpP1 (16). Unambiguous F_o_-F_c_ electron density of 3.0 σ or higher was observed for MAS1-12 at the ClpX/C-binding hydrophobic pocket for 6 out of the 14 subunits (Figs. 4, *A* and *B*, and S4). The determinant of whether a subunit harbored electron density for MAS1-12 may have been the stabilization of the C-terminal tail that is adjacent to the ligand-binding pocket by crystal contacts. It should be noted that, for activation, a dipeptide may be needed in conjunction with an ACP/ADEP-like ligand as is seen in mtClpP1P2 heterotetradecamers (38). The hydrophobic pocket that binds MAS1-12 is formed by two neighboring subunits of the heptameric ring (Fig. 4, *C* and *D*). For all six of the sites, the electron densities indicate that the conformation of the triflouromethylpyridine, gem-dimethyl, and sulfonyl moieties of MAS1-12 are similar for all six sites, whereas the pyrazine and chlorobenzene moieties adopt differing conformations due to more solvent exposure and multiple nearby positively charged residues that may interact with the electron-rich Cl atom (Figs. 4, *E*, *F*, and S4).

The MAS1-12 trifluoromethylpyridine moiety interacts with a hydrophobic cavity composed of Phe63 and Tyr91 of one subunit and Leu49 and Phe83 of another (Fig. 4*E*). The sulfonyl moiety is situated between the hydroxyl group of Tyr91, Lys199, and Met52 of the opposing subunit, whereas the gem-dimethyl moiety sits between Ile29 and Phe63. The C-terminal loop of ctClpP2 that includes a mini-helix, α8, is situated such that it acts like a lid toward the hydrophobic pocket and mediates a neighboring positive electrostatic environment with residues Lys197 and Lys199 in addition to residues Arg28 and Lys58 (Fig. 4*F*). This C-terminal lid is also seen in the structure of apo-paClpP2, though authors of the respective study suggest it occludes the hydrophobic pocket and prevents binding (Fig. 2*A*) (43). For the present study, the positively charged surfaces of the protein increase MAS1-12 binding to the pocket due to the negative-bearing charge of MAS1-12’s Cl and F atoms. The chlorobenzene moiety in particular interacts with Arg23, Lys26, Arg28, and Lys58 (Fig. 4, *E* and *F*).

### Comparing ligand-ctClpP2 binding modes to those from *E. coli* and *Neisseria meningitidis*

**Figure 5:**
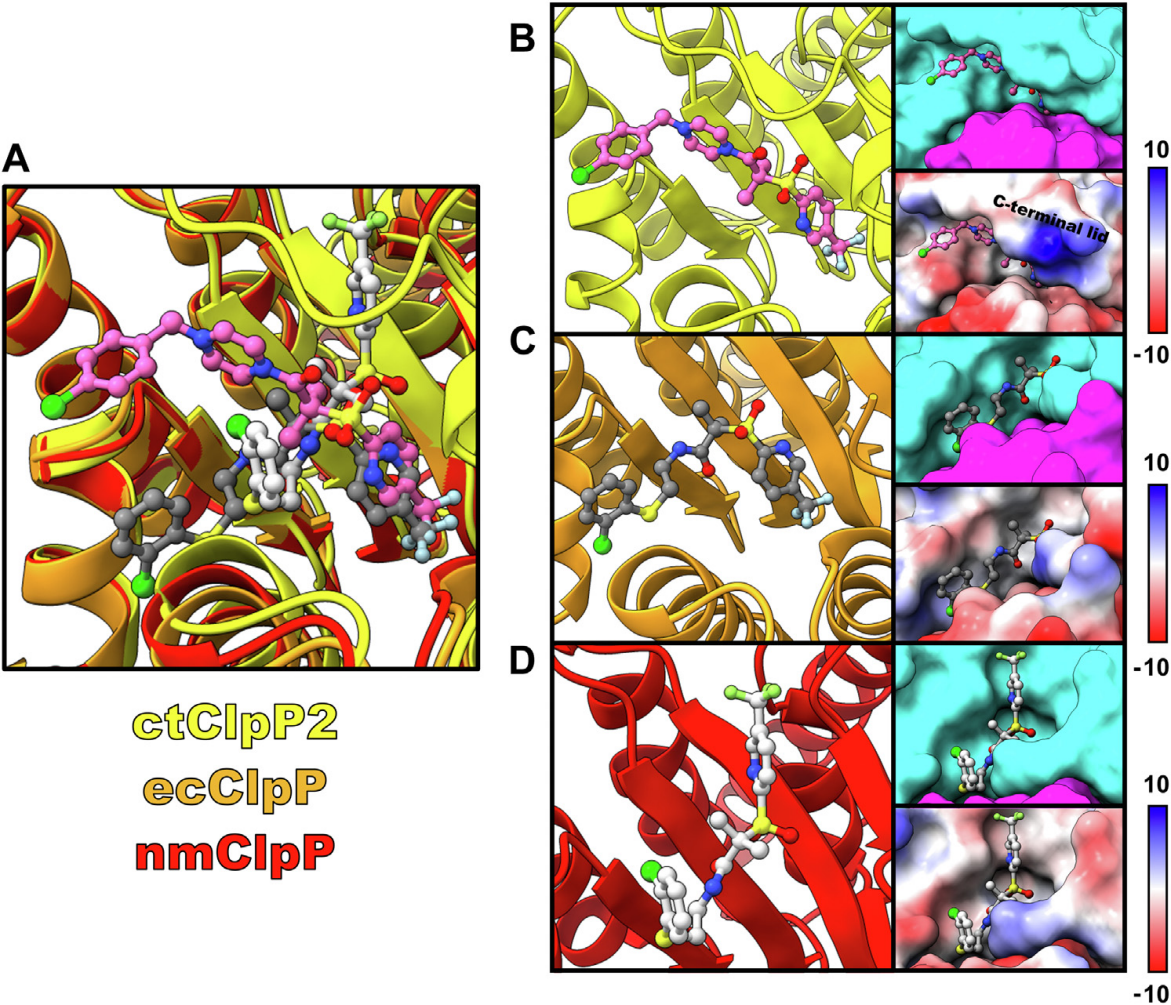
**Comparing ligand binding between ClpP homologs.***A*, superposition of ACP-bound ecClpP and nmClpP to that of MAS1-12–bound (*pink*) ctClpP2. *B*–*D*, individual binding modes where the protein is depicted as ribbons or surfaces. Electrostatic potentials are in units of kcal/(mol·*e*).

The distinguishing structural features of inactive ctClpP2 compared to other homologs prompted us to investigate differences in ligand-binding modes for any available structures that utilize the same class of small molecules. We found two structures, from ecClpP (PDB 6NB1) (46) and *N. meningitidis* (nmClpP; PDB 6W9T) (47), with binding of a molecule with trifluoromethylpyridine, sulfonyl, and gem-dimethyl moieties like the present MAS1-12-bound ctClpP2 structure (Fig. 5*A*). Note that the ACP molecule bound to nmClpP has considerably weaker electron density compared to the ACP molecules bound to ctClpP2 and ecClpP. In contrast with the ecClpP and nmClpP structures that are in the extended, active conformation with aligned catalytic triads, ctClpP2 remains in the compact, inactive conformation.

Close inspection of the structures reveals that in ctClpP2, MAS1-12 binds in a different orientation compared to the ACP molecules bound in ecClpP and nmClpP (Fig. 5*B*). The pyrazine group likely makes MAS1-12 more rigid compared to the other small molecules in ecClpP and nmClpP (Fig. 5, *C* and *D*). A commonality seen between ligand binding in ctClpP2 and ecClpP is the binding of the trifluoromethylpyridine moiety deep within the hydrophobic pocket (Fig. 5, *B* and *C*) where nmClpP lacks this feature (Fig. 5*D*). Analysis of the electrostatic surfaces suggests that the conformation of the trifluoromethylpyridine moiety may be attributed to the electrostatic surfaces of the respective ClpP homolog. For ctClpP2 and ecClpP, the hydrophobic pocket is proximal to a cluster of positively charged surfaces that interact with the electron-rich fluorines of the trifluoromethylpyridine group (Fig. 5, *B* and *C*). Inspection of the electron density for each of the respective structures supports this suggestion, where electron density for the trifluoromethylpyridine moiety in nmClpP is weaker compared to that seen in ctClpP2 and ecClpP (46, 47). Another distinguishing characteristic of ligand-ctClpP2 binding is that a cluster of positive residues at the C-terminal loop of the protein is seen acting as a lid toward the hydrophobic pocket that covers the trifluoromethylpyridine group of MAS1-12 (Fig. 5*B*). Taken together, the comparison of ligand-bound ClpP structures from differing species suggests that ligand-binding modes at the putative unfoldase-binding site is species specific and is determined by the electrostatic surface composition.

### Modeling of ctClpP1

Our study of ctClpP2 led us to survey the structural characteristics of its ortholog, ctClpP1. A model of ctClpP1 was generated using *ALPHAFOLD* structure-prediction software (48). *ALPHAFOLD* implements multiple sequence and pairwise alignment of the input protein sequence and crossreferences alignments with known PDB structures to achieve a predicted 3D structural arrangement of the input sequence. Said another way, the predicted structure of ctClpP1 is built from fragments of PDB structures that have the highest sequence homology.

**Figure 6:**
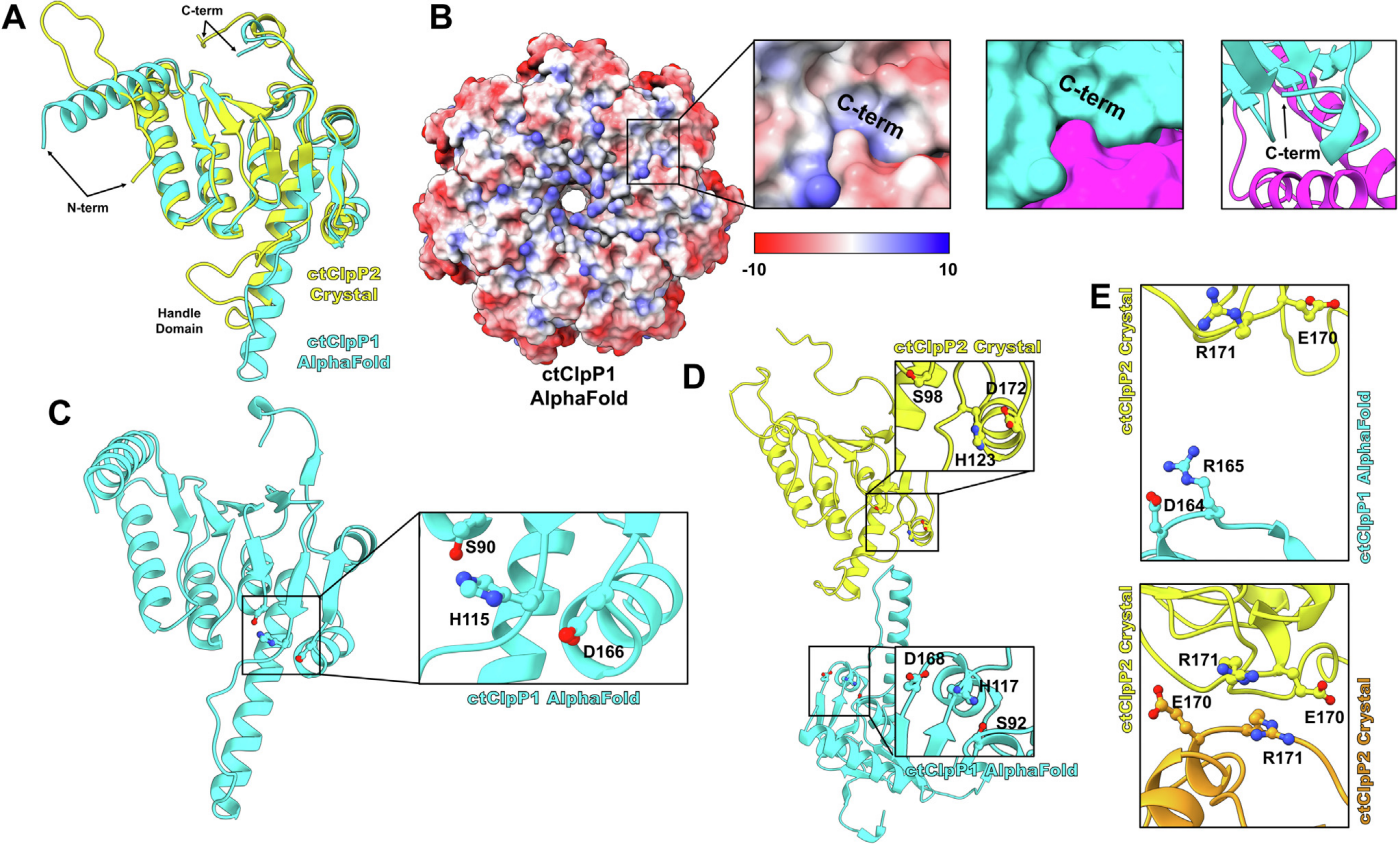
**Modeling of ctClpP1.***A*, comparison of the ctClpP2 crystal structure with the ctClpP1 model generated with *ALPHAFOLD* (48). *B*, predicted apical electrostatic potential surfaces of the ctClpP1 heptamer. Electrostatic potentials are in units of kcal/(mole·*e*). Zoom-ins are one of seven hydrophobic pockets that are occluded by a C-terminal mini-helix. *C*, the predicted ctClpP1 handle domain with an aligned catalytic triad without other activating stimuli. *D*, heterotypic handle domains suggest an incomplete interaction between inactive ctClpP2 and ctClpP1. *E*, comparison of the salt bridge domains that predict oligomerization and proteolytic activity between the predicted heterotypic ctClpP1P2 interaction where ctClpP2 is inactive and observed ctClpP2 homotetradecamers.

For ctClpP2, the structure was distinct from other known homologs by its flexible N-terminal axial loop, a mini-helix at the C terminus, and short handle domain (Fig. 2*A*). The ctClpP1 predicted structure instead has a helical N terminus, a handle domain of similar length to other homologs, and a C-terminal mini-helix (Fig. 6*A*). The N-terminal axial loop is a noted determinant for recognition and activation of paClpP1 and lmClpP2 by their respective ClpX (43), and the lack of the loop in the ctClpP1 suggests ctClpX does not bind ctClpP1. Indeed, ClpX from *C. trachomatis* has been shown to interact with ctClpP2 and not ctClpP1 *in vitro* (18, 30).

ClpP has a C-terminal loop proximal to the hydrophobic pocket that binds the IGF loops of unfoldases and drug inhibitors/activators (33, 44). Both the observed crystal structure of ctClpP2 and the predicted structure of ctClpP1 have loops with C-terminal mini-helices, though the loop of ctClpP1 is shorter in length (Fig. 6*A*). Inspection of the predicted ctClpP1 surfaces indicates that the C terminus occupies the hydrophobic pocket (Fig. 6*B*). The C terminus of ctClpP1, unlike that of ctClpP2, is mostly composed of hydrophobic residues, so the hydrophobic association from the predicted structure is plausible. The occlusion of the binding pocket would prevent the association with the IGF/IGL loops of unfoldases or drug molecules. Like the predicted N terminus of ctClpP1, the predicted C terminus supports the observation that ClpX does not interact with ctClpP1.

Another notable contrast between the ctClpP2 crystal structure and the predicted ctClpP1 structure is the conformational differences of the handle domain (Fig. 6*A*). Based on the ctClpP2 and saClpP structure, the shorter handle domain is attributed to an AA motif that terminates the loop of the handle domain and initiates the α5 helix (Fig. 2, *B*–*D*). For ctClpP1, such a motif is not present and would suggest a handle domain conformation similar to the majority of other ClpP structures that have an α5 helix 2∼3 helical turns longer than ctClpP2 (Fig. 2*A*). Peculiarly, the triad of ctClpP1 is predicted to be aligned in absence of unfoldase recognition and activation (Fig. 6*C*). While this could be attributed to *ALPHAFOLD* sampling triad-aligned homologous structures, such a feature is not unprecedented, as apo paClpP1 is observed with an aligned triad (43).

Whether ctClpP1 and ctClpP2 heptamers may oligomerize into heterotetradecamers *in vivo* is a matter still being investigated. Our previous *in vitro* work demonstrated homotypic and not heterotypic interactions for catalytic activity (18), whereas the work of Pan *et al.* showed *in vitro* protease activity of heterotetradecamers (30). As the studies used different methods to prepare recombinant protein, we wondered if observations between the studies could be attributed to whether inactive ctClpP2 is capable of interacting with ctClpP1. To investigate the possibility of heterotypic interactions in the absence of activated ctClpP2, we analyzed interdigitation between the ctClpP2 crystal structure and the predicted ctClpP1 structure (Fig. 6*D*). Observation of the heterotypic handle domains shows a lack of the intimate interaction (seen in the ctClpP2 homotypic interactions) as a result of differences in handle domain length. An indication of oligomerization and protease activity as seen in other ClpP homologs is the formation of a salt bridge between monomers of opposing heptamers (38, 47, 49). The salt bridge domain neighbors the aspartate of the catalytic triad and is a determinant in the alignment of the catalytic triad but does not necessarily need to be formed for oligomerization. For the ctClpP2 homotetradecamer, the putative salt-bridging residues are within interaction distance, whereas those of the heterotetradecamers are not (Fig. 6*E*). The lack of the salt-bridging interaction is another consequence of differences in handle domain length between orthologs. For the heterotypic salt bridge to form, a conformational change at the handle domain would be needed such as activation of ClpP2 into the extended, active form. Such a change is observed in structures of saClpP where the handle domain gains two helical turns (40). Overall, the predicted ctClpP1P2 heterotetradecamer, where ctClpP2 is inactive, interacts less intimately compared to the ctClpP2 homotetradecamer crystal structure.

### Modeling of active ctClpP2

**Figure 7:**
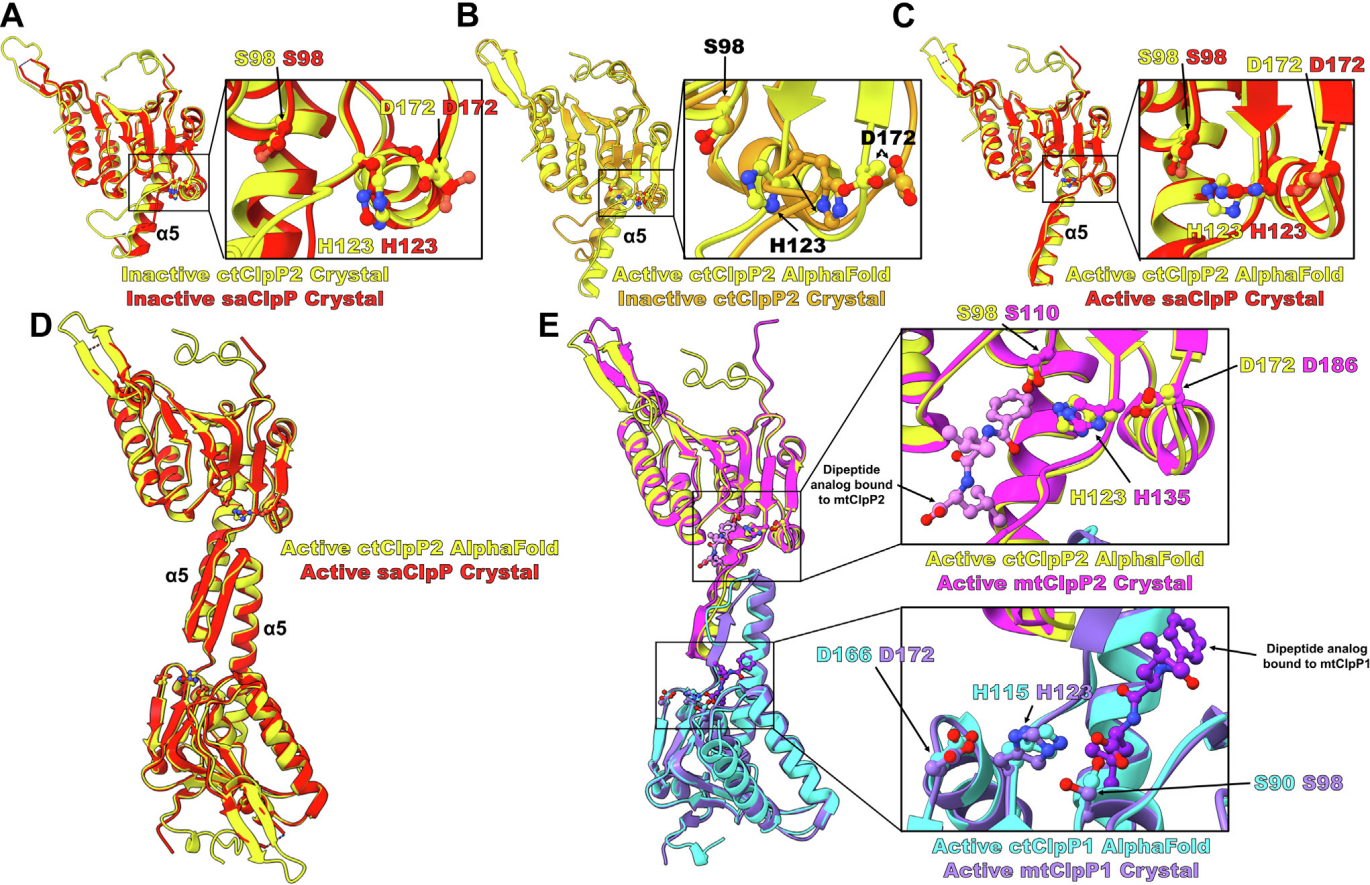
**Modeling of ctClpP2 activation.***A*, comparison of inactive ctClpP2 and saClpP (PDB ID 4EMM) crystal structures. Both structures harbor an unaligned triad. Note that the handle loop of saClpP was not modeled. *B*, modeling of the active ctClpP2 with an aligned triad and comparison with the observed, inactive conformation. The model was generated with *ALPHAFOLD* (48). *C* and *D*, comparison of the active ctClpP2 generated with *ALPHAFOLD* with the active saClpP crystal structure (PDB ID 3V5E). *E*, comparison of the *ALPHAFOLD*-generated models of ctClpP2 and ctClpP1 with that of the dipeptide-bound crystal structure of mtClpP1P2 (PDB ID 5DZK). PDB, Protein Data Bank.

Several observations indicate that ctClpP2 likely undergoes a conformational change to be activated, similar to saClpP, which has not been observed in most other ClpP homologs. First, other inactive ClpP structures coincide with a tetradecameric structure that has ∼10 Å greater height dimensions compared to the present ctClpP2 homotetradecameric crystal structure (Fig. S5) (31, 34, 38, 45, 49, 50). Second, a compact structure like that of ctClpP2 has only been previously observed for saClpP (Fig. 7*A*) (45). Third, for the present ctClpP2 structure, the protein backbone of activity-correlated salt-bridging residues Glu170 and Arg171 are too close for the side chains to interact (Figs. 3*E* and 6*E*) and such a phenomenon has not been observed for other ClpP structures except for saClpP. Fourth, compact saClpP and ctClpP2 share a double-alanine motif that initiates the handle helix (Fig. 2, *C* and *D*). This collection of evidence led us to question the structural nature of the active ctClpP2 form. We utilized *ALPHAFOLD* to sample homologous structures of ClpP in the aligned, active form and constructed a model of ctClpP2 in the active conformation (Fig. 7*B*) (48). Comparing the predicted active ctClpP2 model with the crystal structure of active saClpP yielded a tight RMSD of 0.807 Å across 184 aligned C_α_ pairs (Fig. 7*C*). In conjunction with the inactive crystal structures of saClpP and ctClpP2, the predicted model of active ctClpP2 supports the possibility that ctClpP2 undergoes a dramatic extension of the handle domain for activation. Given the conformational similarity between the observed ctClpP2 and saClpP crystal structures, we hypothesized that should ctClpP2 form active proteolytic homotetradecameric complexes *in vivo*, the complexes would be similar to active saClpP homotetradecameric complexes (Fig. 7*D*).

Since oligomerization of the inactive ctClpP2 with the predicted model of ctClpP1 demonstrated weaker interdigitation interactions compared to the homotypic counterparts (Fig. 6*F*), we next questioned if heterotypic interactions would be feasible among ctClpP2 and ctClpP1 heptamers if ctClpP2 was in the active conformation. We superimposed the active ctClpP1 and ctClpP2 models onto the crystal structure of mtClpP1P2 activated by dipeptide (Fig. 7*E*) (51). The dipeptide-bound mtClpP1P2 crystal structure was chosen because the complex is inactive with only a small molecule activator bound at the ClpX-binding hydrophobic pocket like that of the present inactive ctClpP2 structure (38). Due to this similar allosteric feature, we hypothesized that heterotypic ctClpP2-ctClpP1 interactions would be most similar to that of mtClpP2-mtClpP1 interactions. RMSD comparisons yielded 0.715 Å across 165 aligned C_α_ pairs for ClpP2 and 0.847 Å across 160 aligned C_α_ pairs for ClpP1 (Fig. 7*E*). Inspection of the mtClpP1/P2 active sites shows tight hydrogen bonding among the catalytic triad propagated by the bound dipeptide. It is unclear whether such a dipeptide is needed for activation of ctClpP1P2. Regardless, the predicted active ctClpP1P2 interaction demonstrates remarkable similarity to that of mtClpP1P2. From these data, we conclude that ctClpP2 must undergo a conformational change to achieve functional proteolytic activity in either a homotetradecameric complex or a mixed ctClpP1P2 complex.

## Discussion

The Clp protease systems are key mediators of cytosolic protein degradation in bacteria and serve critical functions ranging from homeostasis to gene regulation to pathogenesis. ClpP is considered a novel therapeutic target to combat different Gram-positive and Gram-negative bacterial infections (50, 52). Design of small molecules to disrupt the ClpP-mediated proteolytic process is an attractive strategy to treat bacterial infections (11, 12). However, for ClpP1 and ClpP2 from *C. trachomatis*, antibiotic design is complicated by a lack of knowledge on the nature of oligomerization, the 3D arrangement of the protein, the dynamics of functional domains, and dissimilarity with homologs (18). In this work, we sought to provide a detailed spatial and functional understanding of ClpP2 from *C. trachomatis* to pursue the design of therapeutic molecules and to better understand the biological function of the chlamydial Clp system.

The mode of oligomerization for ctClpP2 was of particular interest because the functional arrangement of ClpP1–ClpP2 complexes from other species can range from inactive homotypic heptamers, inactive homotypic tetradecamers, active homotypic tetradecamers, or active heterotypic tetradecamers. For ctClpP2, we observed the formation of an inactive homotetradecamer through crystallography. Interestingly, the height dimension is ∼10 Å shorter compared to the tetradecameric structures of lmClpP2, mtClpP1P2, ecClpP, paClpP1, and paClpP2 and suggests a more compact tetradecamer for ctClpP2 (Fig. S5) (43). The shorter dimensions of the ctClpP2 homotetradecamer raised the possibility that the heptamer-heptamer interaction was distinct from most other bacterial homologs.

Fortunately, we were able to resolve residues of the handle and N- and C-terminal domains that have been historically difficult to discern in ClpP structures from other species (Fig. S2) (42). The handle domain is the major interface between heptameric rings and is the vehicle for triad alignment and catalytic activation that is propagated by binding and recognition of the N- and C-terminal regions by the ClpX unfoldase (36, 49). Consequently, understanding the structural features of these domains for the inactive enzyme is of importance for designing activating agents. For ctClpP2, the handle domain has a configuration of isoleucine and leucine residues that leads to hydrophobic packing. Such a hydrophobic interface is not present in other bacterial homologs based on sequence analysis and explains the shorter height dimension of ctClpP2 (Fig. 2). The presence of the atypical interface is supported by the HDX-MS data that shows low deuterium exchange of the ctClpP2 handle domain compared to that of ecClpP (Fig. 3). Indeed, the deuterium exchange rates suggest the ctClpP2 handle domain as having rigidity on par with its secondary structure motifs. These unique handle domain characteristics of ctClpP2 may have implications for catalytic activation and drug design.

The combination of an N terminus axial loop and a C terminus mini-helix adds another layer of distinction for ctClpP2 compared to other homologs. The terminal regions are integral for proteolytic function, where the N-terminal loop interacts with pore-2 loops of ClpX unfoldase and the C-terminal loop mediates binding of the ClpX IGF/IGL loops (33, 44). For ClpPs, the presence of an N-terminal axial loop is required for ClpX recognition, and this region is present in ctClpP2 (Fig. 2*A*) (49). The C-terminal mini-helix is atypical but not unprecedented observation. For paClpP2, a C-terminal mini-helix is also observed, although the terminal residues occlude the hydrophobic pocket with which the ClpX IGF/IGL loops would interact (Fig. 2*A*) (43). The C-terminal helical loop of the ctClpP2 structure consists mostly of electrostatic residues and does not occupy the pocket (Fig. 2*C*). Altogether, the terminal domains of ctClpP2 suggest that an interaction with ctClpX is likely, which is consistent with *in vitro* observations (30).

*C. trachomatis* ClpP1 and ClpP2 contrast with most other bacterial orthologs in that the *clpP1* and *clpP2* genes are located at distinct genomic loci and that the orthologs appear to serve varying functions *in vivo* (26, 27, 28, 29). Of note is that *clpP2* is in the same operon as *clpX*, which strongly supports a ctClpP2-ctClpX interaction. *P. aeruginosa* also exhibits the distinct feature of harboring *clpP1* and *clpP2* at distinct genomic loci with *clpP1* in the same operon as *clpX* (43). Indeed, paClpX has been shown to activate and interact exclusively with paClpP1 where paClpP1 is assembled into either homotetradecamers or heterotetradecamers with paClpP2 *in vivo* (43). The paClpP2 homotetradecamer is inactive, though paClpP2 is required for biofilm formation. Whether assembly into paClpP homotetradecamers or heterotetradecamers occurs is determined by the stage of the *P. aeruginosa* life cycle and leads to differing phenotypes. From inference of the paClpP orthologs, it is plausible that ctClpX activates and interacts exclusively with homotypic ctClpP2 tetradecamers but not homotypic ctClpP1 tetradecamers. Our previous *in vitro* assays suggest such an interaction could be possible since ctClpP2 homotetradecamers exhibited activity, whereas the ctClpP1 counterparts did not (18). Alternatively, and based on our structural data, ctClpX might initially interact with inactive ctClpP2 homotetradecamers to release a heptamer to interact with a ctClpP1 heptamer. This alternative would be consistent with the work of Pan *et al.* who demonstrated ctClpX formed a functional protease with ctClpP1P2 heterotetradecamers (30). It also remains formally possible that, *in vivo*, these scenarios are not mutually exclusive. We have ongoing efforts designed to address these possibilities and whether the composition of the ClpXP protease shifts during the chlamydial developmental cycle. Regardless, our structural data clearly indicate that ctClpP2 can assemble an inactive homotypic tetradecamer. The functional consequences of this require further investigation both *in vitro* and *in vivo*.

Given the unclear structural role of ctClpP1, we took advantage of the most recent developments in structure prediction that interweave evolutionary and spatial information with *ALPHAFOLD* (48) to construct a model of ctClpP1 (Fig. 6*A*). The predicted structure of ctClpP1 suggests a lack of the N-terminal axial loop, a C terminus with a mini-helix that occludes the hydrophobic pocket like paClpP2, and a handle domain length most often observed in other ClpP homologs. The predicted terminal loops are consistent with the structure of paClpP2 and coincide with the absence of ClpX recognition (43). The predicted interaction of the inactive ctClpP2 heptamers with ctClpP1 heptamers leads to the observation that a conformational transition of ctClpP2 would need to occur to stabilize oligomerization of heterotetradecamerization. Specifically, the handle domain of the observed ctClpP2 crystal structure is not long enough to fully interact with ctClpP1. Upon activation, ClpP from *S. aureus* is noted to gain two helical turns at the α5-helix that makes up part of the handle domain (Fig. 7, *A* and *B*) (40). Coincidentally, saClpP tetradecamers have the highest structural similarity to ctClpP2 tetradecamers of the homologs compared in the present study. The comparison with saClpP leads to the suggestion that, if a heterotypic ctClpP2-ctClpP1 interaction were to occur, then ctClpP2 activation precedes hetero-oligomerization.

We observed electron density for a bound small molecule, MAS1-12, in ctClpP2 at the expected binding site for the IGF/IGL loops of ClpX. While activation of ctClpP2 would have been expected when analyzing drug-bound crystal structures of ClpP homologs (49), we observed a misaligned triad and thus the inactive form of ctClpP2 (Fig. 4). This is consistent with our *in vitro* work that demonstrates activation of ecClpP by MAS1-12 but not ctClpP2 or ctClpP1 (16). A similar phenomenon has been observed in mtClpP1P2, where ADEP is observed binding, but the protein structure is that of the inactive form (38). Activation of the heterotetradecamer was only seen with the addition of peptide analogs that bind next to the catalytic triad of both mtClpP1 and mtClpP2. However, conversion of the mtClpP1P2 heterotetradecameric inactive form to the active form by dipeptide binding is largely initialized by mtClpP1, where the dipeptide disrupts the hydrophobic packing of the handle loop to allow catalytic triad alignment (38). This comparison is noteworthy because the handle loop of mtClpP1 has hydrophobicity almost to the same extent as ctClpP2 (Fig. 2*D*). Said another way, a dipeptide activator, in addition to the ACP-like MAS1-12, may be needed for ctClpP1P2 heterotetradecamer or ctClpP2 homotetradecamer activation.

**Figure 8:**
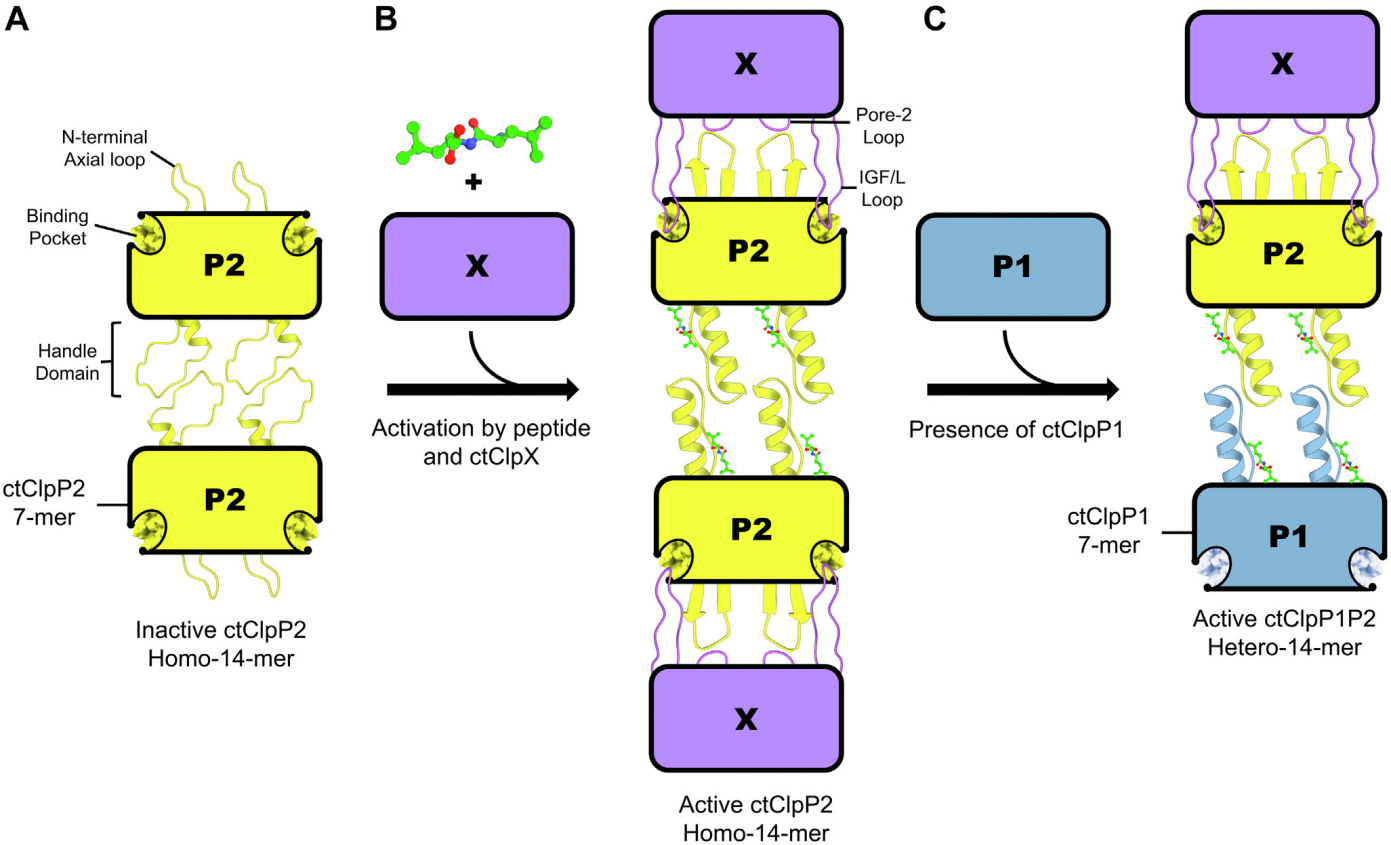
**Model of activation for ctClpP2.***A*, inactive ctClpP2 forms a compacted tetradecamer where the handle domains of one heptamer are compacted against those of the other through hydrophobic interactions. *B*, for alignment of the catalytic triad and proteolytic activation, ctClpX and dipeptide binding are required. The dipeptide binds at the handle domain, disrupts hydrophobic packing, and propagates a conformational change that extends the handle domain and aligns the triad. The ctClpX ATPase binds ctClpP2 through interactions of the ctClpX IGF/L loops and pore-2 loops with the N-terminal axial loops and hydrophobic binding pockets of ctClpP2, respectively. *C*, for the formation of an activated ctClpP1P2 heterotetradecamer, ctClpP2 with an extended handle domain is needed. Protomers of ctClpP1 are unable to interact with ctClpX due to a lack of N-terminal axial loops.

In conjunction with previous *in vitro* studies from our group and others (16, 17, 18, 30, 53) and the architecture of *C. trachomatis* genetic loci (26, 27, 28, 29) in comparison to other orthologous systems (Fig. 7), our new structural insights led us to consider the mode of ctClpP2 activation by ctClpX and interaction with ctClpP1. From the present inactive ctClpP2 crystal structure and our past *in vitro* work (17, 18), we have determined that ctClpP2 is capable of homotypic tetradecameric interactions (Fig. 8*A*). While contrary to the *in vitro* work of Pan *et al.* (30), atomic force microscopy and transmission electron microscopy likewise suggest a homotypic ctClpP2 tetradecamer and reveal dimensions that are too small to suggest ecClpP contamination. Consistent across *in vitro* studies is that ctClpX interacts with ctClpP2 and our ctClpP2 crystal structure reveals the presence of N-terminal axial loops required for ctClpX interaction. For alignment of the triad and proteolytic activity, dipeptide binding at the handle domain may be needed in addition to ctClpX interaction (Fig. 8*B*). The dipeptide is hypothesized to rigidify the handle domain and tighten hydrogen bonding among the catalytic triad as seen in mtClpP1 and mtClpP2 structures (22, 25, 26, 38). As the handle domain of ctClpP2 is also compact like that of saClpP (Fig. 7*A*), catalytic activation of ctClpP2 probably coincides with a large conformational change at the handle domain that extends the homotetradecamer approximately 10 Å (Fig. 7*B*). This ctClpP2 homotypic catalytic activation would be consistent with other ClpP systems that express *clpX* and a single *clpP* gene within the same operon (43, 45).

In the case of an activated ctClpP1P2 heterotetradecameric complex observed *in vitro* by Pan *et al*. (30), there are several indications that ctClpP2 requires handle domain extension before the formation of heterotypic ctClpP1P2 tetradecamers (Fig. 8*C*). First, an inactive ctClpP1P2 heterotetradecamer is not supported by our *in vitro* studies as well as those of Pan *et al*. who suggest inactive ctClpP1 is heptameric (18, 30). Second, the double AA motif found in the handle domain of saClpP and ctClpP2 that correlate with the compact conformation in homotetradecamers is instead an AR motif for ctClpP1 (Fig. 2*D*). The presence of a larger arginine residue would interfere with compaction between a mixed, inactive ctClpP1-ctClpP2 tetradecamer. Third, the analogous system of *P. aeruginosa* that also has ClpP1 and ClpP2 at distinct genetic loci has only had activated heterotetradecamers observed (43). Altogether, the collection of evidence suggests that the formation of activated ctClpP1P2 heterotetradecamers is preceded by activated ctClpP2. We cannot exclude that binding of ctClpX to ctClpP2 may activate a ctClpP2 heptamer to allow for direct association with a ctClpP1 heptamer, which would bypass the formation of an inactive ctClpP2 homotetradecamer.

Altogether, our crystallographic data discerned the spatial arrangement of domains involved in catalytic activation while the HDX-MS provided insight into the dynamics of these domains. Binding of the ACP-like ligand MAS1-12 suggested another stimulus is needed for ctClpP2 activation. *ALPHAFOLD* structure prediction provided the potential structure of ctClpP1 and a model for ctClpP1/2 heterotetradecamers. We expect these results will aid with the design of active antichlamydial agents and will also help unravel the proteolytic mechanism of the ctClpP system.

## Experimental procedures

### Protein expression

*E. coli* BL21(DE3) was transformed using a pLATE vector (Thermo Fisher Scientific) encoding a previously described His-tagged *C. trachomatis* ClpP2 construct (18). Cultures were grown in Lysogeny broth containing 0.1 mg/ml ampicillin. Flasks were shaken at 170 RPM at 37 °C until an *A*_600_ of 0.7. Cultures were then induced with 0.5 mM IPTG, rapidly cooled to 18 °C by immersion in ice water and then shaken at 150 RPM at 18 °C overnight. Cells were pelleted by centrifugation at 4000*g* for 30 min and stored at −20 °C until purification.

### Protein purification

A cell pellet was thawed and resuspended in lysis buffer (25 mM Tris pH 7.0, 150 mM NaCl, 10 mM imidazole, and 2 mM 2-mercaptoethanol) with a protease inhibitor cocktail (P8849, Sigma–Aldrich). Cells were lysed by three passes through an Emulsiflex C3. Cell lysate was clarified *via* centrifugation at 40,000*g* for 30 min and the supernatant filtered using a 0.45 μm filter (EMD Millipore). Protein was purified from the clarified lysate using a cobalt HiTrap^tm^ Chelating HP 5 ml column (GE Healthcare) and an ÄKTApure (GE Healthcare). The column was rinsed with 5 CV of lysis buffer, then the clarified lysate loaded onto the column, and the column was rinsed again with 10 CV of lysis buffer. A 20 CV elution gradient from 10 to 1000 mM imidazole was utilized. Peak fractions were tested using SDS-PAGE, and fractions containing the ClpP2 protein were pooled and concentrated before further purification with size exclusion chromatography using a Superdex 200 16/600 pg column (GE Healthcare). Size exclusion chromatography fractions were examined by SDS-PAGE and the cleanest were tested for monodispersity by dynamic light scattering using a Protein Solutions DynaPro MS/X (Wyatt). Data were interpreted using Dynamics 6.7.6 software (Wyatt Technology) and fractions with a polydispersity <20% were pooled.

### Transmission electron microscopy

A sample of ctClpP2 at a concentration of 0.02 mg/ml in oligomerization buffer (300 μM in 10 mM MgCl2, 100 mM KCl, 25 mM Hepes, 10% glycerol, pH 7.3) was applied on a formvar/silicone monoxide-coated 200 mesh copper grid. The sample was allowed to adhere for 5 min before blotting with absorbent paper and staining with NanoVan negative stain for 30 s. The images were recorded manually using a FEI Tecnai G2 Spirit TEM (FEI; Hillsboro, Oregon) in the UNMC electron microscopy core facility.

### Atomic force microscopy imaging

Freshly cleaved mica was modified with 1-(3-aminopropyl)-silatrane as previously described (54, 55). Protein sample was diluted in oligomerization buffer and deposited onto the piece of 1-(3-aminopropyl)-silatrane mica. The sample was incubated for 2 min, rinsed briefly with several drops of deionized water, and dried with a gentle flow of argon. Images were collected with PeakForce mode with MultiMode Nanoscope 8 system (Bruker Instruments) in tapping mode at ambient conditions. Silicon probes RTESPA-300 (Bruker Nano Inc) with a resonance frequency of ∼300 kHz and a spring constant of ∼40 N/m were used for imaging at a scanning rate of about 1 Hz. Images were processed using the FemtoScan software package (Advanced Technologies Center).

### Synthesis of MAS1-12

This compound was synthesized and denoted as compound 40 in our previous work (16).

### Crystallization

Prior to concentrating the protein, the small molecule MAS1-12 was mixed with ctClpP2 protein at a molar ratio of 2:1 (small molecule: protein) and the mixture was incubated on ice for 20 min. Protein was concentrated to 15 mg/ml in 25 mM Tris, 150 mM NaCl, 10% glycerol, and 2 mM 2-mercaptoethanol using a VIVASPIN 20 PES 10,000 MWCO concentrator (Sartorius) and mixed 1 to 1 (2 μl total volume) on a glass coverslip with a reservoir solution containing 46.6% v/v pH 7.0 Tacsimate (Hampton Research) and 0.5 mM polyoxotungstate [TeW_6_O_24_]^6-^ (TEW) (Jena Biosciences). The coverslip was inverted and sealed over a 500 μl reservoir. Crystals grew within 3 days at room temperature (RT). The crystal was mounted on a 50 μm Micromesh^tm^ (MiTeGen) and soaked in a cryoprotectant solution composed of 100% pH 7.0 Tacsimate (Hampton Research) for 30 s.

### Structure solution and refinement

X-ray diffraction data from a ctClpP2 crystal were measured at a resolution of 2.66 Å at 100 K using a Rigaku FR-E SuperBright Rotating Cu Kα Anode Generator operating at 45 kV and 55 mA with VariMax HR optics and equipped with R-Axis IV++ image plate (Table 1). Data were reduced using *MOSFLM* for indexing and integration (56). Scaling was performed with *AIMLESS* from the *CCP4* software suite (https://www.ccp4.ac.uk/) (57, 58). Due to anisotropic diffraction, unmerged intensities were treated by the *STARANISO* web server (Global Phasing Ltd) to apply an anisotropic diffraction cutoff and anisotropic scaling.

**Table 1:** Data collection and refinement statistics

A. *Data Collection Statistic*	
PDB Code	8DLA
Diffraction source	Rigaku FR-E SuperBright
Detector	R-Axis IV++
Temperature (K)	100
Space group	*P* 1
*a*, *b*, *c* (Å)	96.99, 98.01, 97.97
*α*, *β*, *γ* (°)	97.16, 114.15, 114.16
Wavelengths (Å)	1.5418
No. of images	360
Rotation per image (°)	0.5
Exposure time (s)	480
No. of unique reflections	63,377
Total No. of reflections	121,330
aResolution range (Å)	33.09–2.66 (2.86–2.66)
Multiplicity	1.9 (1.8)
I/σ(I)	5.1 (1.4)
R_merge_	0.079 (0.445)
R_meas_	0.112 (0.629)
R_pim_	0.079 (0.445)
CC_1/2_	0.991 (0.556)
bElliptical data completeness (%)	90.8 (58.0)
bSpherical data completeness (%)	78.2 (20.1)

B. *Refinement Statistics*	

R_work_	0.174
R_free_	0.210
Twin fraction	0.49
Twin operator	-h-l-k
No. of atoms	
Protein	21,588
Ligands	224
Solvent	470
r.m.s. deviations	
Bond lengths (Å)	0.006
Bond angles (°)	1.15
Average *B*-factor (Å^2^)	
Protein	43.99
Ligand	82.51
Solvent	30.19

a Values of the highest resolution shell are given in parentheses.

b Completeness is defined as the measure of the number of actually observed reflections relative to the number of reflections expected. For anisotropic data, only reflections within the ellipsoid that characterizes anisotropic diffraction would ever be observable. For isotropic data, reflections that are observable would be within a spherical shell. Comparing elliptical and spherical data completeness demonstrates the extent of anisotropy. The elliptical cutoff was determined by the *STARANISO* web server from Global Phasing Ltd.

The H and L twinning tests of *TRUNCATE* (59) from the *CCP4* suite suggested the presence of pseudomerohedral twinning. The H test was most consistent with twinning fraction estimates of ∼0.48 performed internally by refinement programs *REFMAC5* and *PHENIX.REFINE*, whereas the L test suggested a twin fraction of ∼0.33 (58, 60). Initial attempts to solve the structure in the *C* 1 2 1 space group resulted in poor electron density maps in some regions, and data were reprocessed in the *P* 1 space group. Molecular replacement was performed with *PHASER-MR* from the *PHENIX* suite using a partial model from PDB coordinates 4JCT (ClpP2 from *L. monocytogenes*) that included only secondary structure elements (39). Model building and map fitting was performed using *COOT*, and iterations of refinement were completed with both *REFMAC5* and *PHENIX.REFINE* using the twin operator -h-l-k (58, 60, 61, 62). Twinned refinement yielded more interpretable maps compared to refinement with untwinned data or data processed in space group *C* 1 2 1 and revealed electron density that was otherwise absent at the catalytically relevant regions of the N-terminal, C-terminal, handle, and binding pocket regions. To aid in model building throughout the structure solution process, feature-enhanced maps were calculated from 2|*F*_o_| - |*F*_c_| density maps using *PHENIX.FEM* from the *PHENIX* software package (63, 64). The method has been shown to strengthen weak signal, reduce model bias and noise, and provide anisotropic corrections (63). Refinements statistics for each of the MAS1-12 ligands are provided in Table S1. The final model was validated with *MOLPROBITY* (65).

### HDX-MS

ctClpP2 protein stock solution in oligomerization was diluted to 30 μM in the same buffer made in 90% D_2_O (pD was corrected for equivalent pH of 7.3) and incubated at RT. Four microliter aliquots were removed at various times of incubation between 15 s and 8 h. The exchange was quenched by the addition of 18 μl of cold 200 mM ammonium phosphate buffer, pH 2.3. Each sample was immediately injected onto the HPLC loop. Enzyme digestion and peptide desalting were started immediately by online injection at a flow rate of 30 μl/min through a pepsin column (Waters Enzymate BEH Pepsin Column; 2.1 × 30 mm, 5 μm) connected to a peptide trap reversed-phase C8 column (Zorbax C8 BW 1 cm × 0,32 mm, 3 μm, 120 A AcuTech Scientific), using 0.05% TFA solution for 5 min. To separate the resultant peptides, a reversed-phase column was used (ZM-C18BW-10000, 5 cm × 1 mm, 3 μm, 120 A, AcuTech Scientific), and peptides were eluted using the following gradient of solution A (0.05% TFA) into solution B (0.05% TFA in acetonitrile): 0 min, 2% to 5% B; 5% to 50% B in 3 min, 50% to 60% B in 8 min, 60% B for 5 min. The column was washed at 80% B for 5 min and re-equilibrated at 2% B for 15 min. All peptides elute during the first 15 min of the gradient. The experiment was done one time. The percent of deuteration was calculated relative to the theoretical maximum deuterium incorporated on the protein. We assume that the first two residues of any peptide did not contain deuterium (or was lost during data analysis) and no deuterium is found from proline residues. Further details in data acquisition are found in the following reference (66).

### Structure prediction

A model of ctClpP1 and ctClpP2 was generated using *ALPHAFOLD* that assembles predicted structures using neural network routines that include spatial and evolutionary information (48). *ALPHAFOLD* is trained to predict a structure most likely to appear as a PDB structure to near experimental accuracy. The measure of confidence given by the structure prediction is given by the local difference distance test (lDDT) for each Cα. The bulk of the predicted structures scored lDDT values above 95%, whereas the N-terminal handle and C-terminal loops scored above 80% (Fig. S6). Several residues of the N-terminal region scored between 50% to 80%.

## Data availability

All obtained data are contained within the article. The raw (primary) data are available upon request (Dr Martin Conda-Sheridan, University of Nebraska Medical Center, email: martin.condasheridan@unmc.edu).

## Supporting information

The supporting information is available free of charge.

It includes supplementary Figures S1–S6.

**Accession Codes**.

8DLA.

## Conflict of interests

The authors declare that they have no conflicts of interest with the contents of this article.

## References

[1] Paavonen J., Eggert-Kruse W. (1999). Chlamydia trachomatis: impact on human reproduction. Hum. Reprod. Update.

[2] Control C.f.D., Prevention (2017).

[3] Sandoz K.M., Rockey D.D. (2010). Antibiotic resistance in chlamydiae. Future Microbiol..

[4] Yang S., Traore Y., Jimenez C., Ho E.A. (2019). Autophagy induction and PDGFR-β knockdown by siRNA-encapsulated nanoparticles reduce chlamydia trachomatis infection. Sci. Rep..

[5] Good J.A.D., Kulén M., Silver J., Krishnan K.S., Bahnan W., Núñez-Otero C. (2017). Thiazolino 2-pyridone amide isosteres as inhibitors of Chlamydia trachomatis infectivity. J. Med. Chem..

[6] Gallegos K.M., Taylor C.R., Rabulinski D.J., Del Toro R., Girgis D.E., Jourha D. (2019). A synthetic, small, sulfated agent is a promising inhibitor of Chlamydia spp. infection *in vivo*. Front. Microbiol..

[7] Abdelrahman Y.M., Belland R.J. (2005). The chlamydial developmental cycle. FEMS Microbiol. Rev..

[8] Skipp P.J., Hughes C., McKenna T., Edwards R., Langridge J., Thomson N.R. (2016). Quantitative proteomics of the infectious and replicative forms of Chlamydia trachomatis. PLoS One.

[9] Ostergaard O., Follmann F., Olsen A.W., Heegaard N.H., Andersen P., Rosenkrands I. (2016). Quantitative protein profiling of Chlamydia trachomatis growth forms reveals defense strategies against tryptophan starvation. Mol. Cell Proteomics.

[10] Gottesman S., Maurizi M.R. (1992). Regulation by proteolysis: energy-dependent proteases and their targets. Microbiol. Mol. Biol. Rev..

[11] Leung E., Datti A., Cossette M., Goodreid J., McCaw S.E., Mah M. (2011). Activators of cylindrical proteases as antimicrobials: identification and development of small molecule activators of ClpP protease. Chem. Biol..

[12] Sass P., Josten M., Famulla K., Schiffer G., Sahl H.-G., Hamoen L. (2011). Antibiotic acyldepsipeptides activate ClpP peptidase to degrade the cell division protein FtsZ. Proc. Natl. Acad. Sci. U. S. A..

[13] Moreira W., Ngan G.J., Low J.L., Poulsen A., Chia B.C., Ang M.J. (2015). Target mechanism-based whole-cell screening identifies bortezomib as an inhibitor of caseinolytic protease in mycobacteria. mBio.

[14] Mundra S., Thakur V., Bello A.M., Rathore S., Asad M., Wei L. (2017). A novel class of Plasmodial ClpP protease inhibitors as potential antimalarial agents. Bioorg. Med. Chem..

[15] Conlon B.P., Nakayasu E.S., Fleck L.E., LaFleur M.D., Isabella V.M., Coleman K. (2013). Activated ClpP kills persisters and eradicates a chronic biofilm infection. Nature.

[16] Seleem M.A., Wood N.A., Brinkworth A.J., Manam S., Carabeo R.A., Murthy A.K. (2022). *In vitro* and *in vivo* activity of (Trifluoromethyl)pyridines as anti-Chlamydia trachomatis agents. ACS Infect. Dis..

[17] Wood N.A., Blocker A.M., Seleem M.A., Conda-Sheridan M., Fisher D.J., Ouellette S.P. (2020). The ClpX and ClpP2 orthologs of Chlamydia trachomatis perform discrete and essential functions in organism growth and development. mBio.

[18] Wood N.A., Chung K.Y., Blocker A.M., Rodrigues de Almeida N., Conda-Sheridan M., Fisher D.J. (2019). Initial characterization of the two ClpP paralogs of Chlamydia trachomatis suggests unique functionality for each. J. Bacteriol..

[19] Flynn J.M., Neher S.B., Kim Y.-I., Sauer R.T., Baker T.A. (2003). Proteomic discovery of cellular substrates of the ClpXP protease reveals five classes of ClpX-recognition signals. Mol. Cel..

[20] Robinson J.L., Brynildsen M.P. (2015). An ensemble-guided approach identifies ClpP as a major regulator of transcript levels in nitric oxide-stressed Escherichia coli. Metab. Eng..

[21] Thompson M.W., Maurizi M.R. (1994). Activity and specificity of Escherichia coli ClpAP protease in cleaving model peptide substrates. J. Biol. Chem..

[22] Raju R.M., Unnikrishnan M., Rubin D.H., Krishnamoorthy V., Kandror O., Akopian T.N. (2012). Mycobacterium tuberculosis ClpP1 and ClpP2 function together in protein degradation and are required for viability *in vitro* and during infection. PLoS Pathog..

[23] Stanne T.M., Pojidaeva E., Andersson F.I., Clarke A.K. (2007). Distinctive types of ATP-dependent Clp proteases in cyanobacteria. J. Biol. Chem..

[24] Stephens R.S., Kalman S., Lammel C., Fan J., Marathe R., Aravind L. (1998). Genome sequence of an obligate intracellular pathogen of humans: chlamydia trachomatis. Science.

[25] Ollinger J., O'Malley T., Kesicki E.A., Odingo J., Parish T. (2012). Validation of the essential ClpP protease in Mycobacterium tuberculosis as a novel drug target. J. Bacteriol..

[26] Akopian T., Kandror O., Raju R.M., UnniKrishnan M., Rubin E.J., Goldberg A.L. (2012). The active ClpP protease from *M. tuberculosis* is a complex composed of a heptameric ClpP1 and a ClpP2 ring. EMBO J..

[27] Dahmen M., Vielberg M.T., Groll M., Sieber Stephan A. (2015). Structure and mechanism of the caseinolytic protease ClpP1/2 heterocomplex from *Listeria monocytogenes*. Angew. Chem. Int. Edition.

[28] Balogh D., Dahmen M., Stahl M., Poreba M., Gersch M., Drag M. (2017). Insights into ClpXP proteolysis: heterooligomerization and partial deactivation enhance chaperone affinity and substrate turnover in *Listeria monocytogenes*. Chem. Sci..

[29] Hall B.M., Breidenstein E.B.M., de la Fuente-Núñez C., Reffuveille F., Mawla G.D., Hancock R.E.W. (2017). Two isoforms of Clp peptidase in *Pseudomonas aeruginosa* control distinct aspects of cellular physiology. J. Bacteriol..

[30] Pan S., Malik I.T., Thomy D., Henrichfreise B., Sass P. (2019). The functional ClpXP protease of Chlamydia trachomatis requires distinct clpP genes from separate genetic loci. Sci. Rep..

[31] Sowole M.A., Alexopoulos J.A., Cheng Y.Q., Ortega J., Konermann L. (2013). Activation of ClpP protease by ADEP antibiotics: Insights from hydrogen exchange mass spectrometry. J. Mol. Biol..

[32] Gatsogiannis C., Balogh D., Merino F., Sieber S.A., Raunser S. (2019). Cryo-EM structure of the ClpXP protein degradation machinery. Nat. Struct. Mol. Biol..

[33] Fei X., Bell T.A., Jenni S., Stinson B.M., Baker T.A., Harrison S.C. (2020). Structures of the ATP-fueled ClpXP proteolytic machine bound to protein substrate. Elife.

[34] Felix J., Weinhäupl K., Chipot C., Dehez F., Hessel A., Gauto D.F. (2019). Mechanism of the allosteric activation of the ClpP protease machinery by substrates and active-site inhibitors. Sci. Adv..

[35] Alexopoulos J., Ahsan B., Homchaudhuri L., Husain N., Cheng Y.-Q., Ortega J. (2013). Structural determinants stabilizing the axial channel of ClpP for substrate translocation. Mol. Microbiol..

[36] Liu K., Ologbenla A., Houry W.A. (2014). Dynamics of the ClpP serine protease: a model for self-compartmentalized proteases. Crit. Rev. Biochem. Mol. Biol..

[37] Brotz-Oesterhelt H., Beyer D., Kroll H.P., Endermann R., Ladel C., Schroeder W. (2005). Dysregulation of bacterial proteolytic machinery by a new class of antibiotics. Nat. Med..

[38] Vahidi S., Ripstein Z.A., Juravsky J.B., Rennella E., Goldberg A.L., Mittermaier A.K. (2020). An allosteric switch regulates Mycobacterium tuberculosis ClpP1P2 protease function as established by cryo-EM and methyl-TROSY NMR. Proc. Natl. Acad. Sci. U. S. A..

[39] Zeiler E., List A., Alte F., Gersch M., Wachtel R., Poreba M. (2013). Structural and functional insights into caseinolytic proteases reveal an unprecedented regulation principle of their catalytic triad. Proc. Natl. Acad. Sci. U. S. A..

[40] Geiger S.R., Bottcher T., Sieber S.A., Cramer P. (2011). A conformational switch underlies ClpP protease function. Angew. Chem. Int. Ed. Engl..

[41] Kimber M.S., Yu A.Y., Borg M., Leung E., Chan H.S., Houry W.A. (2010). Structural and theoretical studies indicate that the cylindrical protease ClpP samples extended and compact conformations. Structure.

[42] Yu A.Y., Houry W.A. (2007). ClpP: a distinctive family of cylindrical energy-dependent serine proteases. FEBS Lett..

[43] Mawla G.D., Hall B.M., Carcamo-Oyarce G., Grant R.A., Zhang J.J., Kardon J.R. (2021). ClpP1P2 peptidase activity promotes biofilm formation in Pseudomonas aeruginosa. Mol. Microbiol..

[44] Kim Y.I., Levchenko I., Fraczkowska K., Woodruff R.V., Sauer R.T., Baker T.A. (2001). Molecular determinants of complex formation between Clp/Hsp100 ATPases and the ClpP peptidase. Nat. Struct. Biol..

[45] Ye F., Zhang J., Liu H., Hilgenfeld R., Zhang R., Kong X. (2013). Helix unfolding/refolding characterizes the functional dynamics of Staphylococcus aureus Clp protease. J. Biol. Chem..

[46] Mabanglo M.F., Leung E., Vahidi S., Seraphim T.V., Eger B.T., Bryson S. (2019). ClpP protease activation results from the reorganization of the electrostatic interaction networks at the entrance pores. Commun. Biol..

[47] Binepal G., Mabanglo M.F., Goodreid J.D., Leung E., Barghash M.M., Wong K.S. (2020). Development of antibiotics that dysregulate the neisserial ClpP protease. ACS Infect. Dis..

[48] Jumper J., Evans R., Pritzel A., Green T., Figurnov M., Ronneberger O. (2021). Highly accurate protein structure prediction with AlphaFold. Nature.

[49] Mabanglo M.F., Houry W.A. (2022). Recent structural insights into the mechanism of ClpP protease regulation by AAA+ chaperones and small molecules. J. Biol. Chem..

[50] Ye F., Li J., Yang C.G. (2016). The development of small-molecule modulators for ClpP protease activity. Mol. Biosyst..

[51] Li M., Kandror O., Akopian T., Dharkar P., Wlodawer A., Maurizi M.R. (2016). Structure and functional properties of the active form of the proteolytic complex, ClpP1P2, from Mycobacterium tuberculosis. J. Biol. Chem..

[52] Moreno-Cinos C., Goossens K., Salado I.G., Van Der Veken P., De Winter H., Augustyns K. (2019). ClpP protease, a promising antimicrobial target. Int. J. Mol. Sci..

[53] Seleem M.A., Rodrigues de Almeida N., Chhonker Y.S., Murry D.J., Guterres Z.D.R., Blocker A.M. (2020). Synthesis and antichlamydial activity of molecules based on dysregulators of cylindrical proteases. J. Med. Chem..

[54] Shlyakhtenko L.S., Gall A.A., Filonov A., Cerovac Z., Lushnikov A., Lyubchenko Y.L. (2003). Silatrane-based surface chemistry for immobilization of DNA, protein-DNA complexes and other biological materials. Ultramicroscopy.

[55] Lyubchenko Y.L., Gall A.A., Shlyakhtenko L.S. (2014). Visualization of DNA and protein-DNA complexes with atomic force microscopy. Methods Mol Biol.

[56] Battye T.G., Kontogiannis L., Johnson O., Powell H.R., Leslie A.G. (2011). iMOSFLM: a new graphical interface for diffraction-image processing with MOSFLM. Acta Cryst. D.

[57] Evans P. (2006). Scaling and assessment of data quality. Acta Cryst. D.

[58] Winn M.D., Ballard C.C., Cowtan K.D., Dodson E.J., Emsley P., Evans P.R. (2011). Overview of the CCP4 suite and current developments. Acta Cryst. D.

[59] Yeates T.O. (1997). Detecting and overcoming crystal twinning. Met. Enzymol..

[60] Afonine P.V., Grosse-Kunstleve R.W., Echols N., Headd J.J., Moriarty N.W., Mustyakimov M. (2012). Towards automated crystallographic structure refinement with phenix.refine. Acta Cryst. D.

[61] McCoy A.J., Grosse-Kunstleve R.W., Adams P.D., Winn M.D., Storoni L.C., Read R.J. (2007). Phaser crystallographic software. J. Appl. Crystallogr..

[62] Emsley P., Cowtan K. (2004). Coot: model-building tools for molecular graphics. Acta Cryst. D.

[63] Afonine P.V., Moriarty N.W., Mustyakimov M., Sobolev O.V., Terwilliger T.C., Turk D. (2015). Fem: feature-enhanced map. Acta Cryst. D.

[64] Adams P.D., Afonine P.V., Bunkoczi G., Chen V.B., Echols N., Headd J.J. (2011). The Phenix software for automated determination of macromolecular structures. Methods.

[65] Chen V.B., Arendall W.B., Headd J.J., Keedy D.A., Immormino R.M., Kapral G.J. (2010). MolProbity: all-atom structure validation for macromolecular crystallography. Acta Cryst. D.

[66] Artigues A., Nadeau O.W., Rimmer M.A., Villar M.T., Du X., Fenton A.W. (2016). Protein structural analysis *via* mass spectrometry-based proteomics. Adv. Exp. Med. Biol..

[67] Sievers F., Wilm A., Dineen D., Gibson T.J., Karplus K., Li W. (2011). Fast, scalable generation of high-quality protein multiple sequence alignments using Clustal Omega. Mol. Syst. Biol..

[68] Waterhouse A.M., Procter J.B., Martin D.M., Clamp M., Barton G.J. (2009). Jalview Version 2—a multiple sequence alignment editor and analysis workbench. Bioinformatics.

