# Supplementary material for: The structure of caseinolytic protease subunit ClpP2 reveals a functional model of the caseinolytic protease system from Chlamydia trachomatis
Source: J Biol Chem. 2022 Dec 1;299(1):102762. doi: 10.1016/j.jbc.2022.102762 (PMC9823225; doi:10.1016/j.jbc.2022.102762)
Supplement: Supplemental data 1 [file mmc1.docx]

Supporting Information

**The structure of caseinolytic protease subunit ClpP2 reveals a functional model of the caseinolytic protease system from *Chlamydia trachomatis***

Jahaun Azadmanesh,^1^ Mohamed A. Seleem,^2^ Lucas Struble,^1^ Nicholas A. Wood,^3^ Derek J. Fisher,^4^ Jeffrey J. Lovelace,^1^ Antonio Artigues,^5^ Aron Fenton,^5^ Gloria E. O. Borgstahl,^1^ Scot P. Ouellette,^3^ Martin Conda-Sheridan^2^

^1^The Eppley Institute for Research in Cancer and Allied Diseases, Fred & Pamela Buffett Cancer Center, University of Nebraska Medical Center, Omaha, NE, 68198

^2^Department of Pharmaceutical Sciences, University of Nebraska Medical Center, 986125, Omaha, NE, 68198

^3^Department of Pathology and Microbiology, University of Nebraska Medical Center, 985900 Nebraska Medical Center, Omaha, NE 68198

^4^School of Biological Sciences, Southern Illinois University Carbondale, Carbondale, IL 62901

^5^Department of Biochemistry and Molecular Biology, The University of Kansas Medical Center, Kansas City, KS 66160

Email: martin.condasheridan@unmc.edu

Phone: +1 402-559-9361

Keywords: *Chlamydia*, ClpP, caseinolytic protease, crystal structure


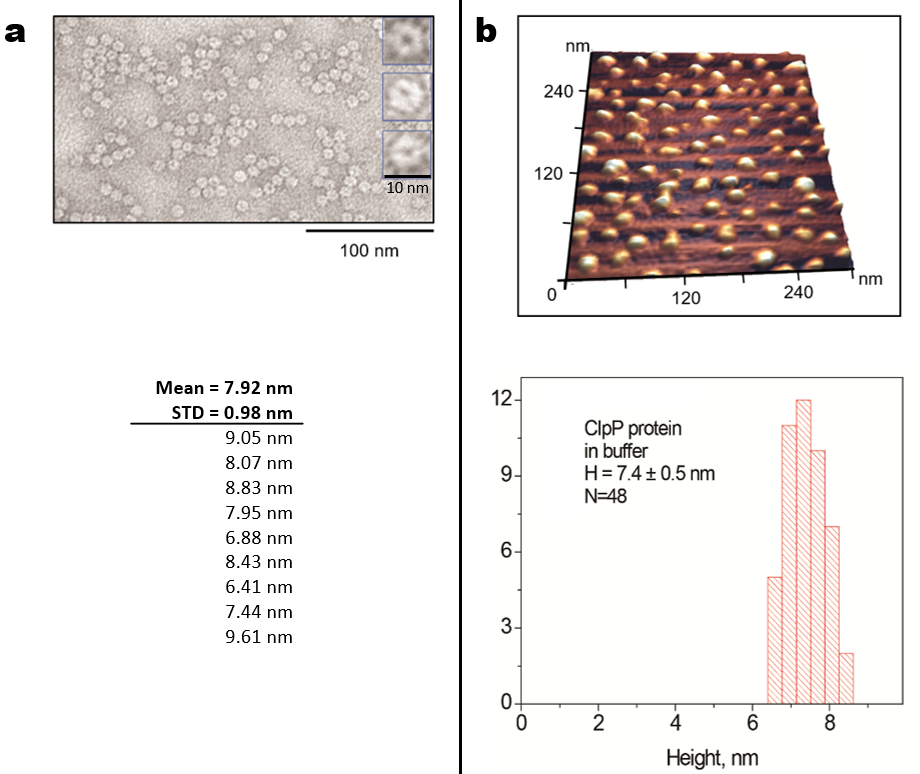


**Supplementary Figure 1. (a)** TEM micrograph of ctClpP2 showing the apical pores and dimensional analysis of the micrographs. **(b)** AFM topological analysis and image showing ctClpP2 morphology on a mica surface.


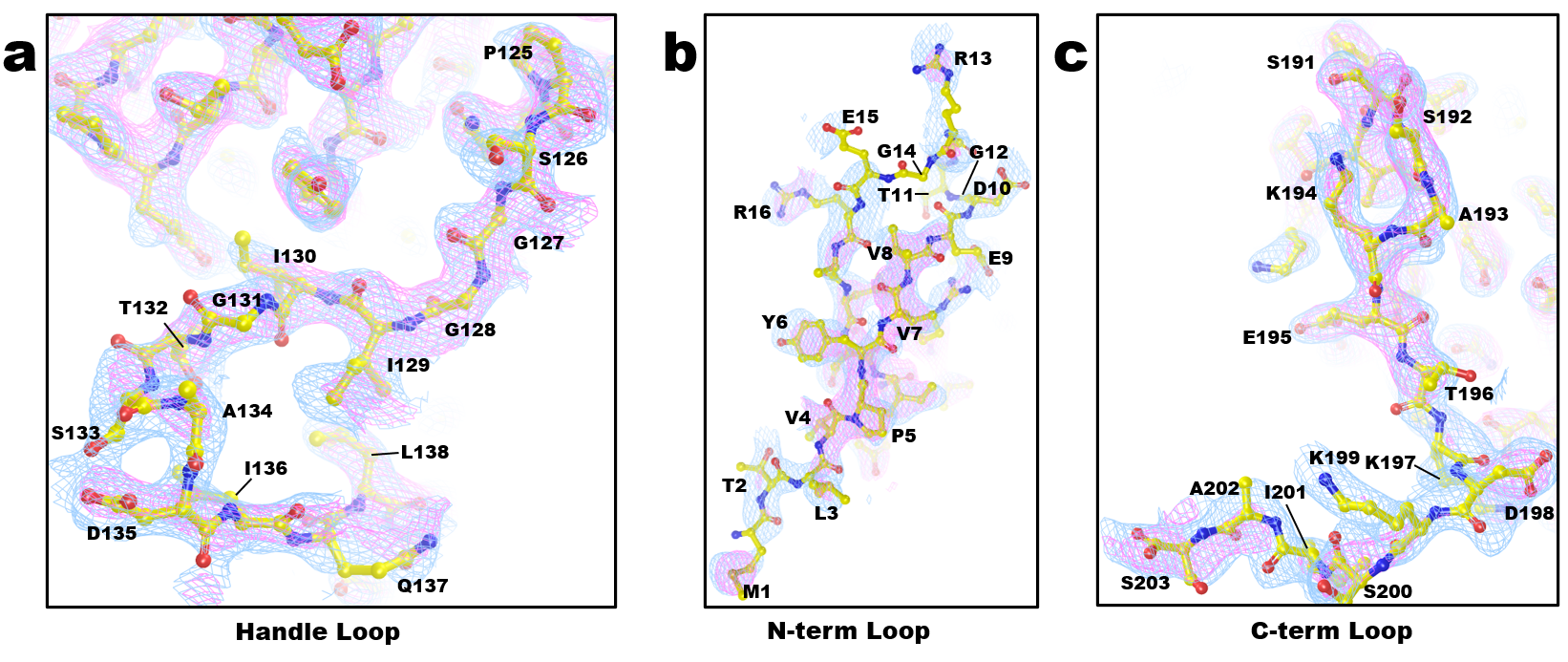


**Supplementary Figure 2.** Representative 2|*F*_o_| - |*F*_c_| (violet) and featured-enhanced (blue) electron density maps of ctClpP2 regions historically difficult to discern in ClpP structures from other species. The 2|*F*_o_| - |*F*_c_| map is contoured at 1.0 σ while the featured-enhanced map is contoured at a level equivalent to 1.0 σ. The feature enhanced map was calculated from the 2|*F*_o_| - |*F*_c_| density map using *PHENIX.FEM* from the *PHENIX* software package (1,2). The method has been shown to strengthen weak signal, reduce model bias and noise, and provide anisotropic corrections (1). (**a**) Handle, (**b**) N-terminal, (**c**) and C-terminal domains expected to be involved in catalytic activation.


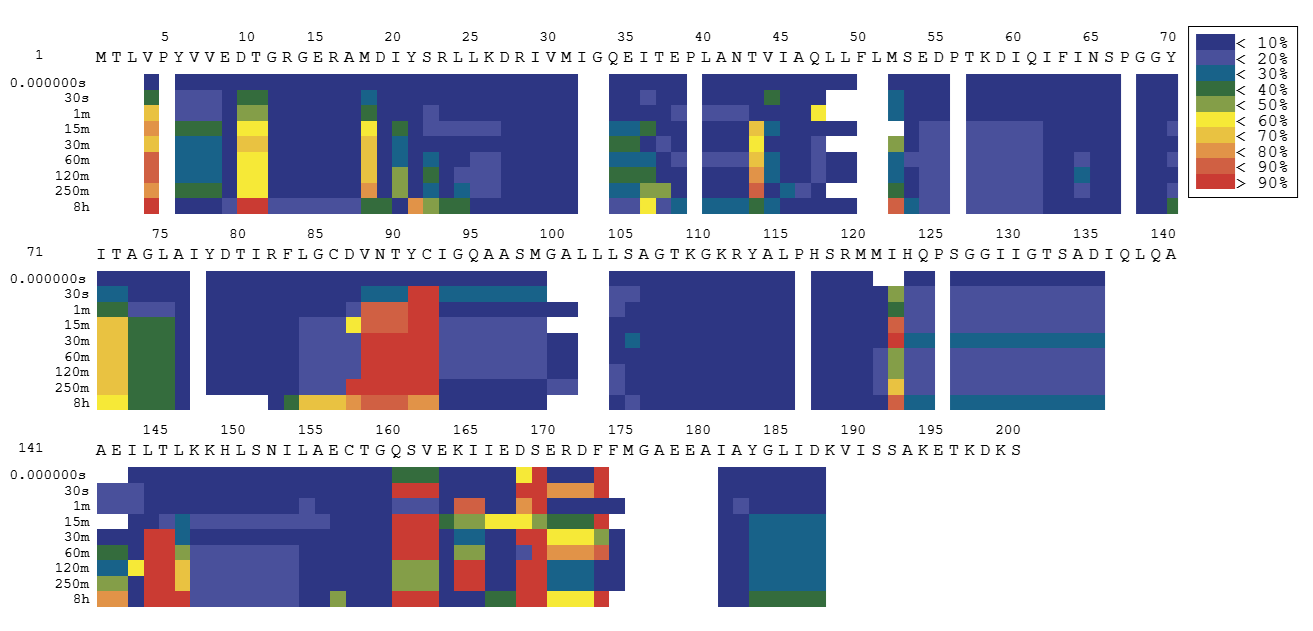


**Supplementary Figure 3.** Heat map of hydrogen-deuterium exchange mass spectroscopy of ctClpP2 at various time points.


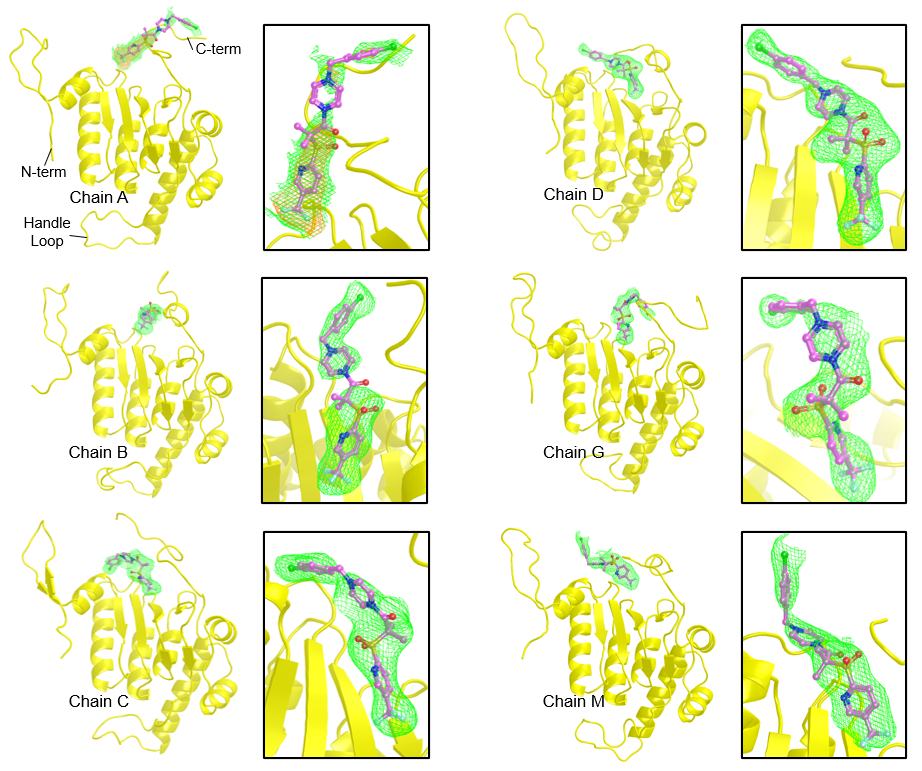


**Supplementary Figure 4.** The binding mode of the ACP-like ligand MAS1-12 to six chains of the ctClpP2 tetradecamer. Green omit |*F*_o_| - |*F*_c_| difference density is contoured at 3.0 σ for all chains. For chain A, orange omit |*F*_o_| - |*F*_c_| difference density contoured at 3.5 σ is also shown since MAS1-12 displays more disorder when binding this chain.


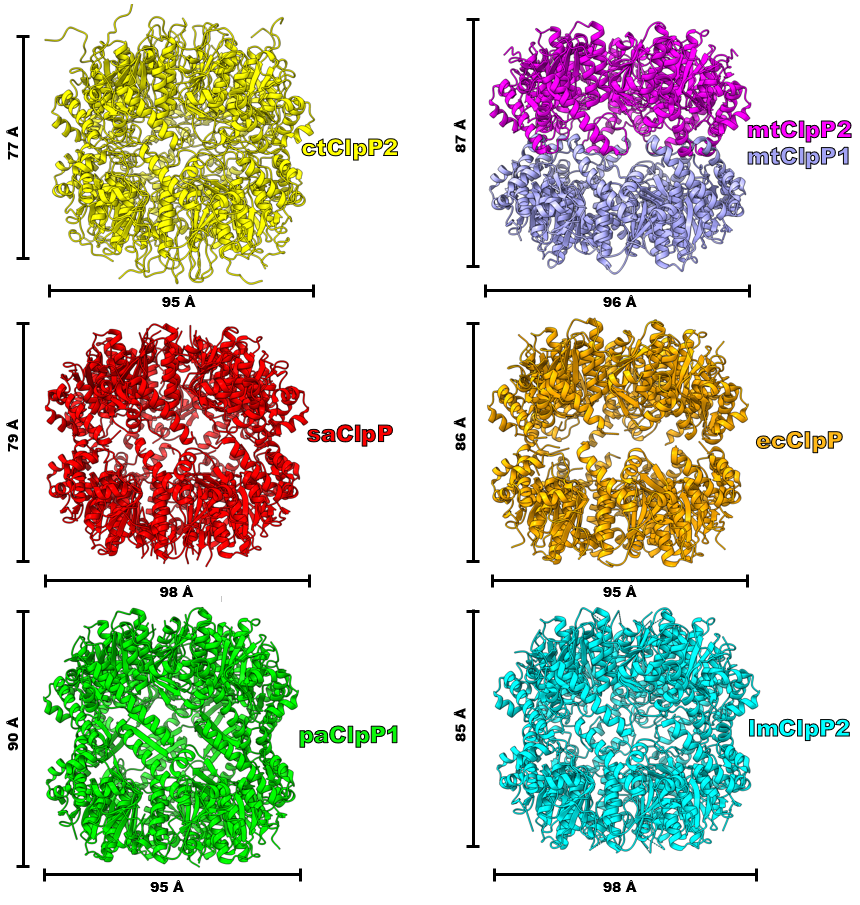


**Supplementary Figure 5.** Comparison of inactive ClpP tetradecamers dimensions from various species: ClpP2 from *Chlamydia trachomatis* (ctClpP2, present study), ClpP2 from *L. monocytogenes* (lmClpP2, PDB ID 4JCT) (3), ClpP from *Staphylococcus aureus* (saClpP, PDB ID 4EMM) (4), ClpP from *E. coli* (ecClpP, PDB ID 3HLN) (5)*¸* heteromeric ClpP1/P2 *M. tuberculosis* (mtClpP1/P2, PDB ID 6VGK) (6), and ClpP1 from *P. aeruginosa* (paClpP1, PDB ID 7M1M) (7). Of note is that paClpP1 resembles an active conformation without activating stimuli such as ClpX or small-molecule binding.


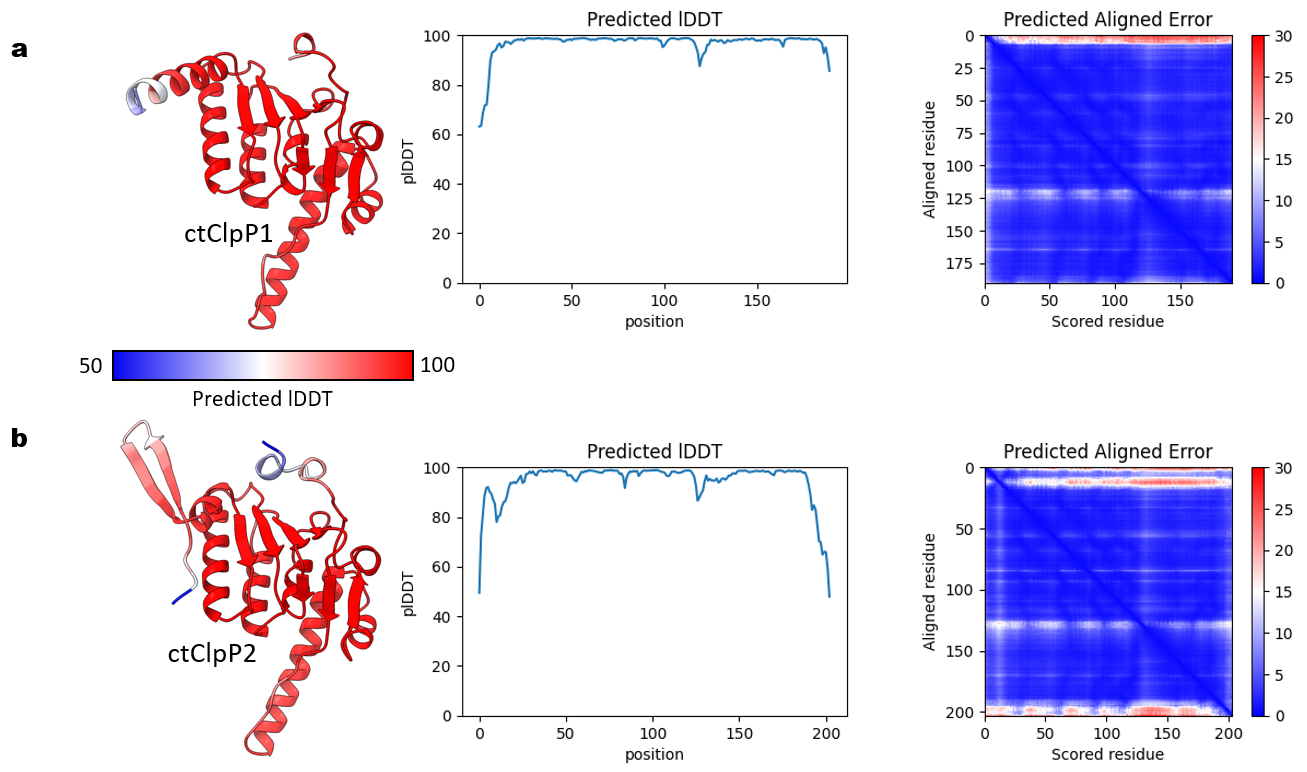


**Supplementary Figure 6.** Structure prediction statistics of (**a**) ctClpP1 and (**b**) active ctClpP2 from *ALPHAFOLD* (8). The measure of confidence given by the structure prediction is given by the local difference distance test (lDDT) for each Cα.

**References**

1. Afonine, P. V., Moriarty, N. W., Mustyakimov, M., Sobolev, O. V., Terwilliger, T. C., Turk, D., Urzhumtsev, A., and Adams, P. D. (2015) FEM: feature-enhanced map. *Acta Cryst. D* **71**, 646-666

2. Adams, P. D., Afonine, P. V., Bunkoczi, G., Chen, V. B., Echols, N., Headd, J. J., Hung, L. W., Jain, S., Kapral, G. J., Grosse Kunstleve, R. W., McCoy, A. J., Moriarty, N. W., Oeffner, R. D., Read, R. J., Richardson, D. C., Richardson, J. S., Terwilliger, T. C., and Zwart, P. H. (2011) The Phenix software for automated determination of macromolecular structures. *Methods* **55**, 94-106

3. Zeiler, E., List, A., Alte, F., Gersch, M., Wachtel, R., Poreba, M., Drag, M., Groll, M., and Sieber, S. A. (2013) Structural and functional insights into caseinolytic proteases reveal an unprecedented regulation principle of their catalytic triad. *Proc. Natl. Acad. Sci. U. S. A.* **110**, 11302-11307

4. Geiger, S. R., Bottcher, T., Sieber, S. A., and Cramer, P. (2011) A conformational switch underlies ClpP protease function. *Angew. Chem. Int. Ed. Engl.* **50**, 5749-5752

5. Kimber, M. S., Yu, A. Y., Borg, M., Leung, E., Chan, H. S., and Houry, W. A. (2010) Structural and theoretical studies indicate that the cylindrical protease ClpP samples extended and compact conformations. *Structure* **18**, 798-808

6. Vahidi, S., Ripstein, Z. A., Juravsky, J. B., Rennella, E., Goldberg, A. L., Mittermaier, A. K., Rubinstein, J. L., and Kay, L. E. (2020) An allosteric switch regulates Mycobacterium tuberculosis ClpP1P2 protease function as established by cryo-EM and methyl-TROSY NMR. *Proc. Natl. Acad. Sci. U. S. A.* **117**, 5895-5906

7. Mawla, G. D., Hall, B. M., Carcamo-Oyarce, G., Grant, R. A., Zhang, J. J., Kardon, J. R., Ribbeck, K., Sauer, R. T., and Baker, T. A. (2021) ClpP1P2 peptidase activity promotes biofilm formation in Pseudomonas aeruginosa. *Mol. Microbiol.* **115**, 1094-1109

8. Jumper, J., Evans, R., Pritzel, A., Green, T., Figurnov, M., Ronneberger, O., Tunyasuvunakool, K., Bates, R., Zidek, A., Potapenko, A., Bridgland, A., Meyer, C., Kohl, S. A. A., Ballard, A. J., Cowie, A., Romera-Paredes, B., Nikolov, S., Jain, R., Adler, J., Back, T., Petersen, S., Reiman, D., Clancy, E., Zielinski, M., Steinegger, M., Pacholska, M., Berghammer, T., Bodenstein, S., Silver, D., Vinyals, O., Senior, A. W., Kavukcuoglu, K., Kohli, P., and Hassabis, D. (2021) Highly accurate protein structure prediction with AlphaFold. *Nature* **596**, 583-589
